# An assessment of the informative value of data sharing statements in clinical trial registries

**DOI:** 10.1186/s12874-024-02168-8

**Published:** 2024-03-09

**Authors:** Christian Ohmann, Maria Panagiotopoulou, Steve Canham, Gerd Felder, Pablo Emilio Verde

**Affiliations:** 1 European Clinical Research Infrastructures Network (ECRIN), Kaiserswerther Strasse 70, 40477 Düsseldorf, Germany; 2 European Clinical Research Infrastructure Network (ECRIN), 75014 Paris, France; 3 European Clinical Research Infrastructure Network (ECRIN), 40764 Langenfeld, Germany; 4 Coordination Centre for Clinical Trials, Heinrich Heine University Düsseldorf, 40225 Düsseldorf, Nordrhein-Westfalen Germany

**Keywords:** Data sharing, Clinical trial registry, Data sharing statement, Individual participant data, Expert, Observer variation

## Abstract

**Background:**

The provision of data sharing statements (DSS) for clinical trials has been made mandatory by different stakeholders. DSS are a device to clarify whether there is intention to share individual participant data (IPD). What is missing is a detailed assessment of whether DSS are providing clear and understandable information about the conditions for data sharing of IPD for secondary use.

**Methods:**

A random sample of 200 COVID-19 clinical trials with explicit DSS was drawn from the ECRIN clinical research metadata repository. The DSS were assessed and classified, by two experienced experts and one assessor with less experience in data sharing (DS), into different categories (unclear, no sharing, no plans, yes but vague, yes on request, yes with specified storage location, yes but with complex conditions).

**Results:**

Between the two experts the agreement was moderate to substantial (kappa=0.62, 95% CI [0.55, 0.70]). Agreement considerably decreased when these experts were compared with a third person who was less experienced and trained in data sharing (“assessor”) (kappa=0.33, 95% CI [0.25, 0.41]; 0.35, 95% CI [0.27, 0.43]). Between the two experts and under supervision of an independent moderator, a consensus was achieved for those cases, where both experts had disagreed, and the result was used as “gold standard” for further analysis. At least some degree of willingness of DS (data sharing) was expressed in 63.5% (127/200) cases. Of these cases, around one quarter (31/127) were vague statements of support for data sharing but without useful detail. In around half of the cases (60/127) it was stated that IPD could be obtained by request. Only in in slightly more than 10% of the cases (15/127) it was stated that the IPD would be transferred to a specific data repository. In the remaining cases (21/127), a more complex regime was described or referenced, which could not be allocated to one of the three previous groups. As a result of the consensus meetings, the classification system was updated.

**Conclusion:**

The study showed that the current DSS that imply possible data sharing are often not easy to interpret, even by relatively experienced staff. Machine based interpretation, which would be necessary for any practical application, is currently not possible. Machine learning and / or natural language processing techniques might improve machine actionability, but would represent a very substantial investment of research effort. The cheaper and easier option would be for data providers, data requestors, funders and platforms to adopt a clearer, more structured and more standardised approach to specifying, providing and collecting DSS.

**Trial registration:**

The protocol for the study was pre-registered on ZENODO (https://zenodo.org/record/7064624#.Y4DIAHbMJD8).

**Supplementary Information:**

The online version contains supplementary material available at 10.1186/s12874-024-02168-8.

## Background

Data sharing (DS) of individual participant data (IPD) in clinical trials has been promoted extensively by different stakeholders and initiatives. Data sharing makes it possible to compare or combine the data from different studies, and to more easily aggregate it for meta-analysis. It allows conclusions to be re-examined and verified or, occasionally, corrected, and it can allow new hypotheses to be tested. Sharing can therefore increase data validity, but it also creates more value from the original research investment, as well as helps to avoid unnecessary repetition of studies [1].

In December 2015, ClinicalTrials.gov added two optional registration fields to the protocol registration and results system (PRS) to address IPD sharing: “Plan to Share IPD” and “Available IPD/Information Type”. In an exploratory study, investigating the use of these fields in ClinicalTrials.gov, potential confusion in the meaning of the terms “IPD” and “sharing”, and inconsistent responses were found in a qualitative analysis [2]. ClinicalTrials.gov added additional subfields to the IPD Sharing Statement section in June 2017 [3]. Currently it includes a primary statement concerning the plan to share IPD (yes, no, undecided), followed by more specific information in case of “yes” (“Plan Description, “IPD Sharing”, “Supporting Information”, “Time Frame”, “Access Criteria” and “URL”). It was expected that by adding additional subfields with greater structure more clear and complete IPD-sharing plans would be provided [2].

In 2017, the International Committee of Medical Journal Editors (ICMJE) required as of 1 July 2018 for manuscripts submitted to ICMJE journals on clinical trials to contain a data sharing statement (DSS) and for clinical trials enrolling participants after 1 January 2019 to include a DSS upon their registration. Individual examples of DSS that fulfil the ICMJE requirements are given [4]. The implementation of ICMJE data-sharing requirements in online journal policies was suboptimal for ICMJE-member journals and poor for ICMJE-affiliated journals [5]. Most trials published in the Journal of the American Medical Association (JAMA), the Lancet, and the New England Journal of Medicine (NEJM) after the implementation of the ICMJE policy declared their intent to make clinical trial data available. However, a wide gap between declared and actual data sharing still exists [6, 7].

The DSS in clinical trial registries have been analysed with respect to willingness to share IPD. Several studies have been performed calculating the percentage of studies with intention to share IPD, sometimes dependent on factors such as geographical location, study type and sponsor type (see Table four in the discussion section). Analysing the supporting information in the DSS of clinical trial registrations allows for the detailed operation and procedure of DS to be assessed. Earlier studies have sometimes found inconsistencies when comparing a generic “yes” for DS with the supplementary text and had to re-classify the assessment after exploring that text. The corrections made were, however, minor and in the range of 1-2% (Table four). What is still missing is an analysis that investigates whether the DSS are providing clear and understandable information for DS that better supports researchers in identifying clinical trials with availability of IPD for secondary use. Such an analysis could answer the following questions:

- Does the text clearly state that IPD sharing is supported?
- Are the conditions/requirements for IPD sharing clearly and understandably formulated?
- Are repositories where the data is to be stored named?
- Are the DSS statements capable of being interpreted, at least in part, by a machine?

We therefore conducted a study with the primary objective of developing and evaluating a classification system for DSS of registered trials, characterising the degree and type of willingness for DS, and the extent and clarity of supporting information. We focused on COVID-19 studies, partly to keep the study within reasonable bounds, partly to ensure the focus was on recent studies, where expectation of a DSS being present are higher, and partly because we were, at the time, involved with other work supporting data sharing in COVID related clinical research. Our secondary objective was to therefore to develop a mechanism to identify COVID-19 trials with a high degree of willingness, and transparent conditions and requirements, for sharing IPD for secondary use.

## Methods

### Eligibility criteria

The following eligibility criteria for selecting clinical trials / clinical studies were used:

- Clinical trials/clinical studies about COVID-19 or SARS-Cov-2.
- Explicit DSS available in the trial registration data.

Both completed and ongoing studies were considered.

### Information sources

The identification of COVID-19 trials was performed with a metadata repository (MDR) developed by the European Research Infrastructure Network (ECRIN). The principal aim of the MDR is to make the data objects generated from clinical research easier to locate, and to describe how each of those data objects can be accessed, providing direct links to them where that is possible.

The central idea is to develop systems that can collect the metadata about the studies and their linked data objects, including object provenance, location, and access details, from a variety of source systems (e.g., trial registries, data repositories, bibliographic systems) and aggregate it into a single repository, the MDR. The MDR standardises the metadata and provides access to it through a single system, accessed via a web portal [8] (https://newmdr.ecrin.org/). The metadata schemas, the data structures, and the data extraction are described in the project’s ‘About’ page [9].

The MDR currently covers the following trial registries:

- ClinicalTrials.gov
- EUCTR
- ISRCTN
- WHO ICTRP (providing access to data from a further 15 national repositories)

In addition, the following repositories and other data sources are covered:

- Pubmed
- BioLINCC
- YODA

Additional file 1 provides some brief further information on the MDR’s information sources. The MDR is updated weekly. As of November 2023, it included metadata from over 800,000 clinical trials / clinical studies and from over 1,300,000 related digital objects. In recent years the number of studies, globally, has grown by about 60,000 studies per year.

### Sample selection

**Fig. 1:**
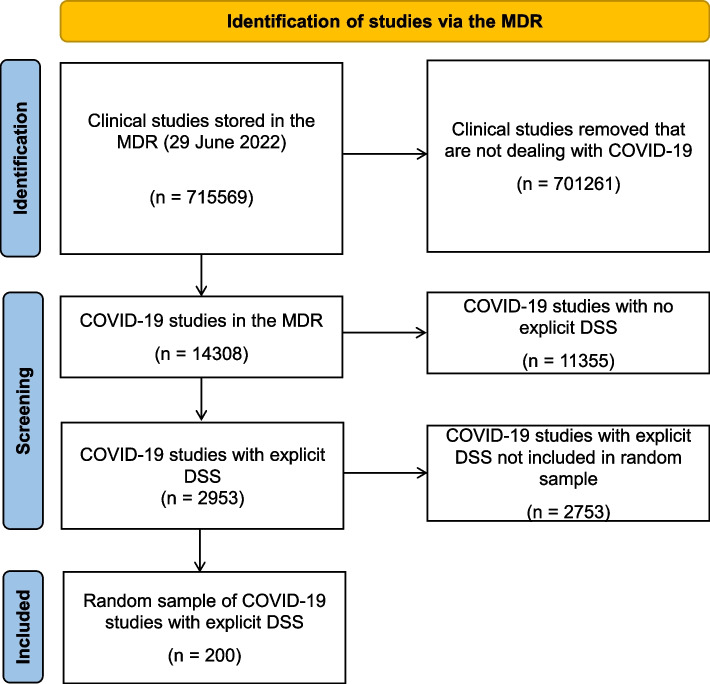
Selection of clinical studies for DSS assessment (according to PRISMA 2020 flow diagram, [10])

The study selection process is summarised in Fig. 1. The initial search was done directly on the underlying data of the MDR, using Structured Query Language (SQL) statements against the database rather than the portal. The search strategy was based on the four terms:

- **'covid',**
- **'coronavirus',**
- **'sars-2'**
- **'sars2'**

The initial selection was of all studies that included one or more of the four terms listed above, either in their titles (all titles were considered) or their related ‘topics’ (associated keywords, including listed target conditions). Then those with an *explicit* DS statement, defined:

- for ClinicalTrials.gov studies, as those that provided more details than the simple initial response of “Yes”, ‘No’ or 'Undecided';
- for data from other registries, as those that had any response at all, as a free text statement

were then identified as suitable for DSS categorisation. The search code is presented in the Additional file 2.

All trials identified as having the COVID-19 linkage criteria, and an explicit DSS (*n*=2,953) were taken into consideration for the study. A subset of the fields extracted from these studies were then extracted into a spreadsheet for easier viewing and analysis. The fields used were:

- Study Id (of the record in the MDR)
- Source Id (a registry Id)
- Id in source (i.e. the trial registry Id)
- Study display (default) title
- Brief description
- DSS

At this stage no attempt was made to distinguish negative from positive DSS. From the full list, a random sample of 200 studies was taken for the study, using the random number generator in Excel. The sample was not stratified according to the length of the DSS – it is a simple random sample from the whole source population.

### Process of DSS assessment and data collection

Two DS experts and one further person (“assessor”) from ECRIN were involved in the study. Information about the experts and the assessor is included in “Author information”.

The three persons independently explored the DSS of the 200 selected studies and provided an assessment according to the following classification (Table 1). Examples were provided to help guide the assessors apply the categories and are also included below, though often with less relevant portions removed to save space. Note that the “(As of <date>)” prefix on many of the statements is added by MDR processing and was not part of the original statement.

**Table 1:** Classification system for DSS (version 1)

**#**	**Unclear**	DSS not understood, and in particular the plans for sharing IPD are not clear or understood.
**Examples**:		
1) “Centers will receive your center-specific evaluation via the publication and via the study groups.” 2) “(As of June 2020): All researchers in this study” 3) “Personal reasons” 4) “The study is under reviewed by medical journal reviewer.”		
**N**	**No sharing**	DSS states or implies that there will be no sharing of IPD. Different specific reasons may be provided.
**Examples**:		
1) “(As of April 2022): We will not plan to share IPD”, 2) “The quantitative dataset will be provided by the service organisation in Leeds and they do not want this data to be made publicly available as it has some commercial sensitivity for them. … … IPD: Not expected to be available”, 3) “Transfer of data to third parties excluded”, 4) “Protect Patient's right EC's policy.”		
**0**	**No plans**	DSS states that there are currently no plans regarding IPD sharing, or a decision has not yet been made
**Examples**:		
1) “(As of September 2021): To be determined”, 2) “(As of December 2021): It is not yet clear, if the data will be shared or not.”, 3) “Need a consensus from colleagues”, 4) “(As of March 2022): There is no default plan to share individual participant data. May be considered upon reaching the VCU IRB”		
**V**	**Vaguely positive**	DSS states or more normally implies that there will be some degree of IPD sharing, but no details are given or sharing appears limited.
**Examples**:		
1) “IPD sharing statement: The current data-sharing plans for this study are unknown and will be available at a later date. IPD: To be made available at a later date”, 2) “(As of July 2020): demographic data of the participants and diagnostic test results will be shared”, 3) “(As of February 2021): Anonymized and pseudonymized data will be published in peer reviewed journals and may be presented at congresses and conferences”, 4) “(As of January 2022): The investigators will be sharing the data, but the management plan is being designed. Information available: Study Protocol”		
**R**	**By request**	DSS implies that IPD can be obtained by request, though who to may not be explicitly stated. If stated it is usually the investigator, sometimes the sponsor.
**Examples**:		
1) “(As of December 2021): Upon request, deidentified participant data collected during the study may be shared. … ... Data will be made available upon request beginning 3 months following publication of the final article from this study, with no end date. … … Data requestors will need to sign a data access agreement.”, 2) “What will be shared: All data can be shared after patients are made unidentifiable. When: Data can be accessible 6 months after results are published… …. Where to obtain: Data can be accessible through an email to the corresponding author. How to obtain: After sending a request email to the corresponding author, data will be sent in 1 month… ….”, 3) “(As of August 2021): Individual data collected during the trial will be available after deidentification upon request to the PI. … …Access Criteria: Deidentified data will be shared with any researcher requesting access. … …” 4) “(As of October 2021): Upon reasonable request, IPD may be shared if fully compliant with HREC requirements and European GRDP regulations”		
**C**	**Complex**	DSS states or implies that IPD will be available but under a more complex regime than any of those described above.
**Examples**:		
1) “De-identified IPD that underlies the results will be shared after publication of primary results and made available indefinitely to investigators whose proposed research have received ethical approval following execution of a data use agreement”, 2) “What will be shared: All data is shareable. When: Access period starts 6 months after the results are published. To whom: It will be available only to researchers working in academic and scientific institutions. Conditions: It is permissible to use this data to find patients who respond better to this treatment. Where to obtain: Contact … … . How to obtain: The request will be answered in a week in consultation with other partners in the project. … …”, 3) “What data will be shared? Outcome data that underlie the results reported in the article (text, tables, figures and appendices) … When will data be available (start and end dates)? Beginning 12 months following analysis and article publication No end date Available to whom? Researchers from a recognised research institution whose proposed use of the data has been ethically reviewed and approved by an independent committee and who accept The University of Melbourne’s conditions for access Available for what types of analyses? Unspecified By what mechanism will data be made available? Written request to principal investigator at … …”, 4) “What will be shared: All related data will be provided upon completion of the study after disidenti?ed (sic) IPD When: The related data will be inde?nitely (sic) provided upon completion of the study. To whom: Relevant information will be provided to all interested parties for humanitarian study and research purposes upon completion of the study. Conditions: On the condition that if any of the study methods (including study design and implementation method, measurement methods, etc.) are used the copyright law be observed and our study be cited. Where to obtain: This will be provided upon completion of the study. Contact: … How to obtain: Contact via email … ”		
**S**	**Via Storage**	DSS states that IPD will be transferred to a repository – may be general, specific or institutional. More often not named specifically.
**Examples**:		
1) “(As of September 2020): The results of this study will be published as open access article and all IPD that underlie results in a publication will be shared, preferentially as supplementary data set or available in a public repository. Any sensitive personal data/information will be not included.”, 2) “General dissemination plan: Planned publication in a high-impact peer-reviewed journal. IPD: Stored in non-publicly available repository”, 3) “(As of February 2022): The fully anonymised datasets analysed during the study will be stored on a publicly available repository. The COG-UK HOCI study to be shared on the UCL Data Repository data-sharing platform so that the data may be reused by other researchers. … …Time frame: This will be done with 6 months of public reporting of results, with data available for 5 years. Access Criteria: Fully open access”, 4) “(As of June 2021): The anonymized database will be uploaded into a public repository. Time frame: The database will be available upon publication of the results. Access Criteria: The data will be available in a public repository …”		

To develop the classification system, a preliminary search had been performed previously for completed COVID-19 trials, again with the support of the MDR. 589 studies were identified with explicit DSS. From these studies 30 were analysed manually by two experts with respect to intention to share IPD from COVID-19 trials. From the analysis, version 1 of the classification of the DSS was derived and used as input for this study.

The process of DSS assessment and data collection consisted of three steps:

- A training session, where the DSS category system, assessment procedure and the data collection process was introduced to the two DS experts and the assessor (virtual meeting).
- Assessment of the DSS, carried out independently by each expert/assessor from home.
- A consensus meeting between the two experts to derive a consensus in case of disagreement (virtual meeting).

A sample size of 200 was selected to give a 95%-confidence interval of around 10% for a given proportion. For a predicted agreement rate between two experts of 80%, the 95%-confidence interval will be between 74% and 86%, using the asymptomatic Wald method.

### Study risk of bias assessment

A risk of biased assessment was not applied in this study.

### Effect measures

The main outcome of the pilot study is the inter-observer variability between two experts in the assessment of the DSS. In addition, the assessment of each individual expert and the assessor is compared.

A further outcome criterion is the agreement between the expert(s) and the consensus (“source of truth”/ “gold standard”).

### Synthesis methods

The bilateral assessments of the two experts and the assessor were tabulated in a cross table and a summary statistic of the agreement/disagreement rate between two persons was generated.

For measuring interrater reliability between the assessment of any two persons the kappa coefficient developed by Fleiss was applied [11], the estimate and the 95%-confidence intervals (CIs) were reported. The statistical analysis was performed using the statistical software R (version 4.2.0).

In cases of disagreement in the categorisation, the two experts from ECRIN met with a third independent person to find a consensus (virtual meeting), preferably by agreement and if not possible, by majority. The result of this consensus process was documented, and the assessment of each expert was compared with the consensus.

### Registration and protocol

The protocol for the study was pre-registered on ZENODO [10] (https://zenodo.org/record/7064624#.Y4DIAHbMJD8). The protocol follows the PRISMA 2020 checklist [12].

## Results

### Comparison between experts

As per the protocol a (virtual) training session was performed, where the assessment of DSS was introduced and demonstrated with examples (9 September 2022). Thereafter, the assessment procedure for the 200 sources began. The assessment was finalised by expert A and B on 19 September and by assessor C on 20 September 2022. Expert A needed 2 hours for assessment, expert B 12 hours and assessor C 6 hours.

Detailed comparisons of the categorisations provided by expert A, expert B and assessor C are provided in Additional file 3. For experts A and B, (Table A1a), the overall agreement is 70% (139/200). The estimated kappa is 0.62 with a 95% CI [0.55, 0.70], clearly significant. The results show that the agreement is in the range of moderate to substantial.

The results are different when the assessment of experts A and B is compared to assessor C,). Overall agreement of assessor C against expert A is 42% (83/200), with an estimated kappa of 0.33 and a 95% CI [0.25, 0.41]. Against expert B the corresponding figures are 44% (88/200), with an estimated kappa of 0.35 and a 95% CI [0.27, 0.43]. In both cases the results clearly indicate the agreement is low. In Additional file 4 an explanatory analysis is presented, where the cross tables have been condensed in different ways.

### Consensus agreement

Two virtual meetings were performed (15 and 16 November 2022) to achieve consensus for the cases where expert A and B disagreed (*n*=61). In contrast to the study protocol, assessor C was not included in these meetings and did not participate in the consensus process. Agreement of assessor C with the experts A and B was low and analysis revealed some systematic deviations, indicating that more training and experience would have been necessary for assessor C to produce comparable results.

The consensus meetings were chaired by an independent clinical research expert (C.O.). In all cases agreement could be achieved between the experts A and B and the chair after discussion of the individual DSS and the assessments by the experts.

The results from the consensus are presented in Table 2:

**Table 2:** Classification of the 200 clinical studies after experts’ consensus

**Classification**	**Specification**	**Number of cases**
Unclear	-	13% (25/200)
No data sharing	-	14% (27/200)
No plans	-	11% (21/200)
Yes	Vague	16% (31/200)
	Defined request conditions	30% (60/200)
	Defined storage location	8% (15/200)
	Complex conditions	11% (21/200)
Total	-	200

The assessment of experts A and B were compared with the consensus result. The results are shown in Additional file 3.Overall agreement for expert A was 87% (173/200), and for expert B it was 82% (163/200). The estimated kappa is 0.83 with a 95% CI [0.78, 0.89] for expert A, and 0.77 with a 95% CI [0.71, 0.84] for expert B. In both cases, these results can be classified as an *almost perfect* level of agreement. The individual assessments of the experts and the consensus are listed in Additional file 5.

After the consensus meetings and the intensive discussion, the classification system, whilst retaining the same categories, was extended with clearer definitions (see Table 3):

**Table 3:** Classification system for DSS (version 2)

**#**	**Unclear**	DSS not understood. Either the statement makes no sense (e.g., is not coherent English), or IPD is not mentioned at all, or IPD is mentioned but the intention to share or not share is not made clear, even in the vaguely positive sense described by ‘vaguely positive’ below. N.B. Responses that indicate that ‘demographic data’ or ‘clinical data’ or similar will be shared are ambiguous – the data could be summary tables rather than IPD. Unless clarified elsewhere in the DSS, therefore, such responses fall into this category. In all cases the intention, or not, to share IPD for secondary use is simply unknown.
**N**	**No sharing**	DSS states or implies that there will be no sharing of IPD. Different specific reasons may be provided. Usually an explicit statement is made (e.g. “transfer of data to third parties excluded”) but sometimes a reference is made to an implied reason for not sharing (“patients’ confidentiality”). DSS that refer *only* to sharing of aggregate or results data, or *only* to publication of a journal paper are also assumed to fall into the ‘no sharing’ category.
**0**	**No plans**	DSS states that there are currently no plans regarding IPD sharing, or a decision has not yet been made. Usually, an explicit statement but may be implicit in statements such “need to consult with collaborators / participants”. N.B. “Not yet planned…” would fall into this category, while “not planned…” would be classified as ‘no sharing’.
**V**	**Vaguely positive**	Vaguely positive. DSS states or implies that there will be some degree of IPD sharing, but no details are given or sharing appears limited, e.g. “To be made available at a later date”. This is the weakest of the positive statements. Sometimes the intention is made clear but the details are not yet available – e.g. “The investigators will be sharing the data, but the management plan is being designed.”
**R**	**By request**	DSS states that IPD can be obtained by request, usually as an explicit statement, occasionally just as an email address. Few further details about the access process are provided and there are no additional prerequisites identified (other than, sometimes, the fact that a Data Use Agreement must be signed). The contact person is usually the investigator, sometimes the sponsor, but the role may not be stated.
**C**	**Complex**	DSS states or implies that IPD will be available and describes or references a more complex regime than a simple request. Common additional requirements are for requests to be reviewed by a data access or trial committee, for the applicants to have an approved protocol, or to follow a named procedure set out in a document or web page. These DSS are therefore positive but provide more detail than simply stating the data can be requested.
**S**	**Via Storage**	DSS explicitly states that IPD will be transferred to a specific data repository – may be general, specific to clinical research or institutional - which would normally be named. The data repository (e.g., CSDR, Vivli, or Yoda) should have its own access policies and procedures, and not be just a repository for publicly available documents (e.g., Zenodo). This is a version of the ‘complex+’ category above, but with the additional requirements partially provided by repository systems.

## Discussion

The willingness to share IPD according to DSS in trial registrations varies between 4.8% and 15.7% [1, 6, 13–18] (Table 4). In a retrospective cohort study performed with the Australian New Zealand Clinical Trials Registry (ANZTR), the commitment to share data was 22% [7]. A study with African study registers revealed nearly complete willingness to share data, not in line with studies from elsewhere in the world [17].

**Table 4:** Studies investigating the willingness to share IPD in clinical trial registries

**Author, year**	**Time**	**Resource**	**Trial type**	**“yes” in DSS field of trial registration**
Bergeris et al., 2018 [2]	January 2016 to August 2017	CT.gov	Interventional trial	10.9% (2782/25551)
Tan et al., 2021 [7]	December 2018 to November 2019	ANZTR	Interventional trial	22% (329/1517) commitment to share data
Statham et al, 2020 [13]	January 2018 to June 2018	CT.gov	Interventional trial	5.5% (112/23040)
Li et al., 2021 [14]	Before 30 June 2020	CT.gov	COVID-19, interventional trial	15.7% (145/924) 17.3% (159/924) after re-classification
Larsson et al, 2022 [15]	Up to September 2021	CT.gov	COVID-19, interventional trial	15% (417/2759) 15.7% (432/2759) after re-classification
Merson et al., 2022 [16]	January 2019 to December 2020	WHO ICTRP	Registered trial	4.8% (28,684/593,595)
Malinga, et al., [17]	From 2019 to September 2022	PACTR, SANCTR	Registered trial	97% (1763/1818 in PACTR 100% (477/477) in SANCTR
Xu et al., 2020 [18]	Up to 31 December 2018	WHO ICTRP	Global registration of studies sponsored by China	Around 5%

(“yes” in the DDS field “Plan to share IPD (yes, no, undecided)

*DSS* Data sharing statement

We applied a slightly different search algorithm to the MDR on 8 January 2023, this time including all COVID-19 studies with an explicit DSS, but also *including* those who simply said “Yes”, ‘No’ or ‘Undecided’ without providing any further details. This was to obtain a sample more similar to that used by other studies. This provided 7618 trials, of which 6203 were registered in ClinicalTrials.gov. From these trials 16.55% indicated a “yes” in the DSS. This is similar to the studies from Li and Larson [14, 15].

Table 4, summarising existing studies, shows that willingness to share IPD is between around 5 and 20%, so still very low. Our study has shown that even when willingness to share data is formulated, the DSS is vague or not specific in most cases, if the DSS is examined in more detail. So, there is doubt in a significant number of cases, whether declared willingness to share data really lead to active sharing.

In our study we looked at the structure and content of explicit DSS and tried to derive clearer and more specific categories related to DS, helping the data requestor to get a clearer picture of the DS procedure. It turned out that some DSS are not understandable and remain “unclear”, even after close inspection. Some DSS indicate clearly that there is no willingness of data sharing (“no data sharing”) and in some “no plans” could be elucidated (corresponding to “undecided”). With respect to a positive indication of DS, we found a graduation of statements, starting from “yes but vague” via “yes with defined request conditions” to “yes, already with a defined storage location”. Apart from that, there were some DSS, which were formulated in a way that the concrete conditions of DS could not be uniquely identified by experts. These cases were put into a category “complex”.

It had been hoped that with the help of the classification system the proportions of DSS expressing willingness for DS, supported by clear and understandable criteria for potential users, could be clarified. Before a classification system can be applied to create new knowledge, however**,** it should be validated. In our case we decided to involve DS experts, let them apply the classification system to a random sample of DSS and then have a look at the degree of observer variation. It turned out that there was moderate to substantial agreement between the experts (kappa=0.62, overall agreement=70%), indicating that the system could be of value when applied by DS experts. Agreement considerably decreased when these experts were compared with a third person, less experienced and trained in data sharing (the “assessor”). Here the agreement was low (kappa=0.33/0.35, overall agreement= 42%/44%). This indicates that without adequate training and experience specific in data sharing terminologies, the classification system will be of limited value. It seems to be that for non-expert users, DSS may be interpreted with substantial variability and uncertainty.

To progress and to apply the classification system, consensus between the two DS experts was sought and taken as the gold standard for further analysis. The overall agreement of the experts with the consensus was higher than 80% with almost perfect agreement in the kappa analysis. This is promising but not surprising because the same sample was analysed again, and the results certainly need confirmation in an independent prospective study. Examining the consensus results, we found that in 63.5% (127/200) of the cases at least some degree of willingness of DS was indicated. From these statements around one quarter (31/127) were vague, stating or implying that there will be some degree of IPD sharing, but no details are given or sharing appears limited. In around half of the cases (60/127) it was stated that IPD could be obtained by request, usually as an explicit statement, and only in slightly more than 10% of the cases (15/127) it was stated that the IPD would be transferred to a specific data repository. In the remaining cases (21/127), a more complex regime was described or referenced, which could not be allocated to one of the other categories. This analysis shows that even if willingness to DS is declared, the DSS is often of limited help with respect to the concrete conditions and requirements for DS.

Our study shows that trained and experienced experts can classify DSS and filter out the more promising ones for data reuse. It also provides some insight to the research community on the importance of making clear statements with respect to IPD sharing, and the current level of such statements.

The difficulty is, of course, that the volume of clinical research work and publications is such that no collection of experts would ever be able to provide or maintain a comprehensive categorisation of DSS on a manual basis. In the future it may be possible to apply new techniques, similar to the text-mining algorithms that screens biomedical publications and detect cases of open data (ODDPub - Open Data detection in Publications, [19]), and that could be used to assess data sharing rates on the level of subject areas, journals, or institutions.

It would be interesting to see whether the classification system developed could be supported and applied semi-automatically through involvement of natural language processing (NLP) techniques, utilising machine learning (ML) or Artificial Intelligence (AI). Here the consensus developed by the experts might serve as the core of a “gold standard” for comparison with the results from a machine categorisation.

## Conclusions

To achieve an optimal clinical research data sharing environment, both for IPD meta-analysis and other secondary research purposes, it is important that the sources of potential data sharing are identifiable and that the processes required to apply for IPD are clear. To improve transparency and data reuse, journals should promote the use of unique pointers to data set location and standardized choices for embargo periods and access requirements [6]. But the Data Sharing Statements provided by researchers, and found in trial registries as well as journal papers, also need to be clear and easily categorizable, so that researchers can quickly identify potential resources.

The study has shown that, even when DSS are semi-structured, it is currently not easy for even relative experts to clearly and consistently identify what many DSS mean. A substantial portion of DSS are still unclear, vague, or rather complex. In addition, any practical system of classification would need DSS to be machine processable, and that is far from the case at the moment. It would be much more efficient, and some degree of machine processing much more possible, if data providers, data requestors, funders and platforms need to adopt a more ‘joined-up’ and standardised approach [20].

An important initial step towards a clearer and more interpretable DSS is first to require a DSS in all cases. Further steps include being clearer about exactly what information is being requested, improving the structure of the IPD sharing statement and using a standardised and controlled vocabulary for specification of all relevant aspects of the DSS – free text should be minimised. For example, if IPD can be shared by request, the procedure and the institutions involved should be described by selecting adequate options from a pre-defined list.

## Weakness of the study

A weakness of the present study is the limited sample size investigated (*n*=200), which leaves some uncertainty in the conclusions. Nevertheless, the results demonstrate the deficiencies in the current handling of DSS in clinical trial registrations, which needs to be improved.

Focusing on COVID 19 related studies may have given an overly positive impression of current data sharing intentions and statements, because of the intense interest in, and substantial encouragement of, data sharing during and immediately after the pandemic. Research in other areas of clinical research is likely to show less intention to share IPD, but further work would be needed to confirm this.

In addition, the experts agreeing on the consensus and applying the classification system were the same and no different data sets were used for both exercises, possibly leading to bias. Here, a prospective independent study is needed to confirm the results.

### Supplementary Information

**Additional file 1.** MDR Information Sources.

**Additional file 2.** Data extraction and SQL statements.

**Additional file 3.** Detailed comparisons.

**Additional file 4.** Exploratory analysis.

**Additional file 5.** Individual assessment and consensus.

## Data Availability

The distribution of the assessments of the DSS by the experts (anonymised) is included in the tables. To avoid any problems with data privacy and identification of the experts (who are at the same time named co-authors), the individual raw data related to the classification of DSS are not included in the manuscript.

## Abbreviations

AI: Artificial intelligence
ANZTR: Australian New Zealand Clinical Trials Registry
BioLINCC: Biologic Specimen and data Repository Information Coordinating Center
COVID-19: Coronavirus disease
DS: Data sharing
DSS: Data Sharing Statement
ECRIN: European Clinical Research Infrastructure Network
EUCTR: EU Clinical trials Register
ICJME: International Committee of Medical Journal Editors
ID: Identifier
IPD: Individual participant data
ISRCTN: International Standard Randomised Controlled trial Number
ICMJE: International Committee of Medical Journal Editors
JAMA: Journal of the American Medical Association
MDR: Metadata Repository
NEJM: New England Journal of Medicine
NLP: Natural Laguage Processing
PRS: Protocol registration and results system
SARS-COV-2: Severe Acute Respiratory Syndrome Coronavirus-2
SQL: Structured Query Language
WHO ICTRP: World Health Organization. International Clinical Trials Registry Platform
YODA: Yale University Open Data Access
