# Supplementary material for: An assessment of the informative value of data sharing statements in clinical trial registries
Source: BMC Med Res Methodol. 2024 Mar 9;24:61. doi: 10.1186/s12874-024-02168-8 (PMC10924983; doi:10.1186/s12874-024-02168-8)
Supplement: Supplementary file 1 — Additional file 1. MDR Information Sources. [file 12874_2024_2168_MOESM1_ESM.docx]

**An assessment of the informative value of data sharing statements in clinical trial registries (Ohmann et al.)**

**Additional file 1**

**MDR Information Sources**

Most of the sources used by the MDR are ‘study based’, that is the data is organised by clinical research study, and includes various study attributes and properties, including data on some of the associated data objects. This is the case, naturally enough, for all of the data obtained from trial registries, (which provide the great majority of the MDR’s data) but it also the case for the data obtained from the Yoda and BioLINCC repositories.

The 21 study based data sources are listed below, listed in descending order of size (CTR = Clinical Trial Registry). For each, the total number of studies for which the metadata was stored (in October 2023) stored is given, as well as the method used to obtain the data.

| **Name** | **Studies** | **Data via** |
| --- | --- | --- |
| ClinicalTrials.gov (US) | 466,789 | Direct |
| Chinese CTR | 72,776 | WHO ICTRP |
| Japan Primary Registries Network | 65,824 | WHO ICTRP |
| Clinical Trials Registry - India | 57,599 | WHO ICTRP |
| EU Clinical Trials Register | 43,489 | Direct |
| Iranian Registry of Clinical Trials | 36,704 | WHO ICTRP |
| Australian / New Zealand CTR | 24,422 | WHO ICTRP |
| ISRCTN | 23,955 | Direct |
| Deutschen Register Klinischer Studien | 15,178 | WHO ICTRP |
| Netherlands National Trial Register | 9,822 | WHO ICTRP |
| Clinical Research Information Service (S Korea) | 8,771 | WHO ICTRP |
| Thai CTR | 8,535 | WHO ICTRP |
| Registro Brasileiro de Ensaios Clínicos | 6,555 | WHO ICTRP |
| International Traditional Medicine CTR | 4,223 | WHO ICTRP |
| Pan-African CTR | 3,998 | WHO ICTRP |
| Registro Peruano de Ensayos Clínicos | 2,000 | WHO ICTRP |
| Yoda | 449 | Direct |
| Sri Lanka CTR | 445 | WHO ICTRP |
| Registro Público Cubano de Ensayos Clínicos | 431 | WHO ICTRP |
| BioLINCC (NIH) | 276 | Direct |
| Lebenon CTR | 150 | WHO ICTRP |
|  |  |  |

Note that the total number of studies in the system is less than the total of the amounts from each source, because about 40,000 studies are registered in more than one trial registry.

Some data is obtained directly from the source, by API or file download, but for a large proportion of the sources the data is obtained from a file obtained from the WHO ICTRP, the International Clinical Trials Registry Platform. The WHO receives data on a regular basis from its network of primary registries, that together provide global coverage, and as well as publishing this data itself it also makes it available as a downloadable file.

When trial registration began in the 2000s it was dominated by ClinicalTrials.gov, which registered about 85% of the studies being registered at that time. Other registries that were important at that time were ISRCTN and the European EU CTR. Over recent years, however, one of the significant developments in registration has been the rapid increase in importance of other registries, especially those in China, India, Japan and Iran, as well as smaller registries in South America, Asia and Africa. In recent years ClinicalTrials.gov has registered about 50%, sometimes less, of the total registered globally (data from the MDR), though it remains by far the biggest of the trial registries.

Only two repositories in the list of 21 are not trial registries. Yoda is a repository and managed access service run by Yale university. Most of the material in it has been deposited by the pharma company Johnson & Johnson, or one of its subsidiaries. BioLINCC is the study and material repository maintained by the NIH’s National Heart, Lung and Blood Institute, also in the US, and mostly contains details on the studies funded by that institute or related programs, and the available data objects.

One important source is organised primarily by object: PubMed, providing details on journal papers. There were, in October 2023, just over 250,000 linked ‘PubMed object’s in the system.
