# Supplementary material for: An assessment of the informative value of data sharing statements in clinical trial registries
Source: BMC Med Res Methodol. 2024 Mar 9;24:61. doi: 10.1186/s12874-024-02168-8 (PMC10924983; doi:10.1186/s12874-024-02168-8)
Supplement: Supplementary file 2 — Additional file 2. Data extraction and SQL statements. [file 12874_2024_2168_MOESM2_ESM.docx]

**Additional file 2**

**An assessment of the informative value of data sharing statements in clinical trial registries (Ohmann et al.)**

**Additional file 2**

**Data extraction and SQL statements**

Some of the SQL statements used for the search are presented here. A full set of the SQL statements used, and an explanation of the tables and fields can be obtained by request to the authors.

--********************************************************

-- Create tables of relevant records

--********************************************************

-- code 2.1 Create table of all distinct studies related to covid / sars2

drop table if exists st.cov;

create table st.cov

as

select s.*

from core.study_search ss

inner join core.studies s

on ss.id = s.id

where

(title_lexemes ilike '%covid%'

or title_lexemes ilike '%sars-2%'

or title_lexemes ilike '%coronavirus%'

or title_lexemes ilike '%sars2%'

or topic_lexemes ilike '%covid%'

or topic_lexemes ilike '%sars-2%'

or topic_lexemes ilike '%coronavirus%'

or topic_lexemes ilike '%sars2%');

-- code 2.2 Create table of source studies reporting that they have IPD now

drop table if exists st.cov_ipd;

create table st.cov_ipd

as

select sids.study_id, sids.source_id,

sids.sd_sid, sids.is_preferred,

s.* from core.study_search ss

inner join core.studies s

on ss.id = s.id

inner join nk.study_ids sids

on s.id = sids.study_id

where

(title_lexemes ilike '%covid%'

or title_lexemes ilike '%sars-2%'

or title_lexemes ilike '%coronavirus%'

or title_lexemes ilike '%sars2%'

or topic_lexemes ilike '%covid%'

or topic_lexemes ilike '%sars-2%'

or topic_lexemes ilike '%coronavirus%'

or topic_lexemes ilike '%sars2%')

and has_ipd = true

-- code 2.3 Create table of all distinct studies related to covid / sars2 with non-null / non-empty DSS

drop table if exists st.cov_dss;

create table st.cov_dss

as

select s.*

from core.study_search ss

inner join core.studies s

on ss.id = s.id

where

(title_lexemes ilike '%covid%'

or title_lexemes ilike '%sars-2%'

or title_lexemes ilike '%coronavirus%'

or title_lexemes ilike '%sars2%'

or topic_lexemes ilike '%covid%'

or topic_lexemes ilike '%sars-2%'

or topic_lexemes ilike '%coronavirus%'

or topic_lexemes ilike '%sars2%')

and s.data_sharing_statement is not null

and trim(s.data_sharing_statement) <> ''

Initial checks on the data were performed, using the core MDR dataset on 29/06/2022. These were done to check the consistency of the data between different tables belonging to the MDR.

The ‘study search’ table in the MDR was used in these statements because it includes fields composed of lexemes (words and word stems) constructed by text indexing processing on the MDR data, on both the sets of titles for each study (title_lexemes field) and the sets of keywords (topic_lexemes field). In most cases the keywords are MESH coded, and both the original topic term and the MESH coded version are included within the lexemes list.

The table is rebuilt during each weekly aggregation as an aid to searching against study attributes in the portal, but in this context, it also makes searching against COVID-19 related terms much easier.

In the additional file code provided above:

- 2.1 identifies and extracts the core study data into a new table from those studies that fulfil the COVID-19 linkage criteria (n=**14,308**)
- 2.2 identifies and extracts the core study data into a new table from those studies that fulfil the COVID-19 linkage criteria and which say they have IPD available *now* (n=**4**), while
- 2.3 identifies and extracts the core study data into a new table from those studies that fulfil the COVID-19 linkage criteria and which have a non-null data DSS (n=**2,953**)
