# Supplementary material for: An assessment of the informative value of data sharing statements in clinical trial registries
Source: BMC Med Res Methodol. 2024 Mar 9;24:61. doi: 10.1186/s12874-024-02168-8 (PMC10924983; doi:10.1186/s12874-024-02168-8)
Supplement: Supplementary file 3 — Additional file 3. Detailed comparisons. [file 12874_2024_2168_MOESM3_ESM.docx]

**An assessment of the informative value of data sharing statements in clinical trial registries (Ohmann et al.)**

**Additional file 3**

**Detailed comparisons**

The comparison of the assessment of expert A and B is presented in table A1a.

|  | Expert A | | | | | | | |
| --- | --- | --- | --- | --- | --- | --- | --- | --- |
|  |  | # | N | 0 | V | R | C | S |
| Expert B | # | 11 | 7 | 2 | 0 | 0 | 0 | 0 |
|  | N | 0 | 14 | 2 | 0 | 0 | 0 | 0 |
|  | 0 | 0 | 0 | 17 | 3 | 0 | 0 | 0 |
|  | V | 6 | 5 | 0 | 21 | 2 | 2 | 1 |
|  | R | 1 | 2 | 0 | 5 | 57 | 4 | 0 |
|  | C | 1 | 1 | 0 | 3 | 8 | 8 | 2 |
|  | S | 1 | 0 | 0 | 2 | 0 | 1 | 11 |

**Table 1a: Cross-table of the DSS assessment: Expert A versus expert B** # = unclear, N = no sharing, 0 = no data sharing plan, V = yes but vague, R = defined
request conditions, C = complex, S = defined storage conditions

Overall agreement is 70% (139/200). The estimated kappa is 0.62 with a 95% CI [0.55, 0.70].

|  | Assessor C | | | | | | | |
| --- | --- | --- | --- | --- | --- | --- | --- | --- |
|  |  | # | N | 0 | V | R | C | S |
| Expert A | # | 15 | 0 | 1 | 1 | 1 | 2 | 0 |
|  | N | 9 | 15 | 1 | 3 | 0 | 1 | 0 |
|  | 0 | 1 | 4 | 16 | 0 | 0 | 0 | 0 |
|  | V | 4 | 1 | 8 | 13 | 0 | 6 | 2 |
|  | R | 4 | 1 | 8 | 17 | 9 | 28 | 0 |
|  | C | 2 | 0 | 1 | 1 | 2 | 9 | 0 |
|  | S | 1 | 0 | 0 | 1 | 2 | 4 | 6 |

**Table 1b: Cross-table of the DSS assessment: Expert A versus assessor C** # = unclear, N = no sharing, 0 = no data sharing plan, V = yes but vague, R = defined
 request conditions, C = complex, S = defined storage conditions

Overall agreement is 42% (83/200). The estimated kappa is 0.33 with a 95% CI [0.25, 0.41].

|  | Assessor C | | | | | | | |
| --- | --- | --- | --- | --- | --- | --- | --- | --- |
|  |  | # | N | 0 | V | R | C | S |
| Expert B | # | 16 | 1 | 1 | 1 | 0 | 1 | 0 |
|  | N | 0 | 15 | 0 | 1 | 0 | 0 | 0 |
|  | 0 | 0 | 3 | 17 | 0 | 0 | 0 | 0 |
|  | V | 12 | 0 | 10 | 11 | 1 | 2 | 1 |
|  | R | 4 | 1 | 6 | 19 | 9 | 30 | 0 |
|  | C | 3 | 1 | 1 | 3 | 2 | 13 | 0 |
|  | S | 1 | 0 | 0 | 1 | 2 | 4 | 7 |

**Table 1c: Cross-table of the DSS assessment: Expert B versus assessor C** # = unclear, N = no sharing, 0 = no data sharing plan, V = yes but vague, R = defined
 request conditions, C = complex, S = defined storage conditions

Overall agreement is 44% (88/200). The estimated kappa is 0.35 with a 95% CI [0.27, 0.43].

**Comparisons of expert scores with consensus**

|  | Consensus | | | | | | | |
| --- | --- | --- | --- | --- | --- | --- | --- | --- |
|  |  | # | N | 0 | V | R | C | S |
| Expert A | # | 19 | 1 | 0 | 0 | 0 | 0 | 0 |
|  | N | 5 | 24 | 0 | 0 | 0 | 0 | 0 |
|  | 0 | 1 | 2 | 18 | 0 | 0 | 0 | 0 |
|  | V | 0 | 0 | 3 | 28 | 0 | 1 | 2 |
|  | R | 0 | 0 | 0 | 2 | 58 | 7 | 0 |
|  | C | 0 | 0 | 0 | 0 | 2 | 13 | 0 |
|  | S | 0 | 0 | 0 | 1 | 0 | 0 | 13 |

**Table 2a: Cross-table of the DSS assessment: Expert A versus consensus**# = unclear, N = no sharing, 0 = no data sharing plan, V = yes but vague, R = defined
 request conditions, C = complex, S = defined storage conditions

Overall agreement between expert A and the consensus is 87% (173/200). The estimated kappa is 0.83 with a 95% CI [0.78, 0.89].

|  | Consensus | | | | | | | |
| --- | --- | --- | --- | --- | --- | --- | --- | --- |
|  |  | # | N | 0 | V | R | C | S |
| Expert B | # | 15 | 4 | 1 | 0 | 0 | 0 | 0 |
|  | N | 0 | 16 | 0 | 0 | 0 | 0 | 0 |
|  | 0 | 0 | 0 | 20 | 0 | 0 | 0 | 0 |
|  | V | 6 | 5 | 0 | 24 | 0 | 2 | 0 |
|  | R | 1 | 2 | 0 | 5 | 59 | 2 | 0 |
|  | C | 2 | 0 | 0 | 2 | 1 | 16 | 2 |
|  | S | 1 | 0 | 0 | 0 | 0 | 1 | 13 |

**Table 2b: Cross-table of the DSS assessment: Expert B versus consensus**# = unclear, N = no sharing, 0 = no data sharing plan, V = yes but vague, R = defined
 request conditions, C = complex, S = defined storage conditions

Overall agreement between expert B and the consensus is 82% (163/200). The estimated kappa is 0.77 with a 95% CI [0.71, 0.84].
