# Supplementary material for: An assessment of the informative value of data sharing statements in clinical trial registries
Source: BMC Med Res Methodol. 2024 Mar 9;24:61. doi: 10.1186/s12874-024-02168-8 (PMC10924983; doi:10.1186/s12874-024-02168-8)
Supplement: Supplementary file 4 — Additional file 4. Exploratory analysis. [file 12874_2024_2168_MOESM4_ESM.docx]

**An assessment of the informative value of data sharing statements in clinical trial registries (Ohmann et al.)**

**Additional file 4**

**Exploratory analysis**

As part of an exploratory analysis, the cross-tables (table 2, 3a, 3b) have been condensed in 4 different ways. The results are summarized in the table below. The agreement between expert A and B is above 85% when R is compared to the collapsed rest of the table and R or S is collapsed to the rest. If the cross-table is condensed to V or R or S or C versus the rest (equivalent to IPD to be shared), it is 90%.

The agreement between expert A and assessor C is between 60 and 70% for the different collapsing scenarios, except for collapsing R, S, V and C (corresponding to “IPD to be shared”), here it is around 80%. The agreement between expert B and assessor C is below 60% for the different collapsing scenarios, except for collapsing R, S, V and C (corresponding to “IPD to be shared”), here it is around 70%.

| **Condensing of cross-table** | **Meaning** | **Expert and assessor agreement (%)** | | |
| --- | --- | --- | --- | --- |
|  |  | **A versus B** | **A versus C** | **B versus C** |
| Full cross-table with 7 categories | No collapsing | 70% (139/200) | 42% (83/200) | 37% (74/200) |
| Cross-table condensed to R (yes, no) | indicating that IPD can be obtained by request versus the rest collapsed | 89% (178/200) | 64% 127/200) | 59% 118/200 |
| Cross-table condensed to R or S (yes, no) | indicating that IPD can be obtained by request or IPD is transferred to repository versus the rest collapsed | 86% (171/200) | 66% (131/200) | 57% (113/200) |
| Cross-table condensed to V or R or S (yes, no) | indicating that there is at least some degree of IPD sharing versus the rest collapsed | 81% (162/200) | 64% (127/200) | 53% (106/200) |
| Cross-table condensed to V or R or S or C (yes, no) | indicating that there is at least some degree of IPD sharing versus the rest collapsed, including the complex cases (C) | 90% (180/200) | 81% (162/200) | 71% (141/200) |

**Table: Exploratory analysis with respect to condensing the cross-table (table 1)** V = yes but vague, R = defined request conditions, C = complex, S = defined storage
conditions
