# Supplementary material for: An assessment of the informative value of data sharing statements in clinical trial registries
Source: BMC Med Res Methodol. 2024 Mar 9;24:61. doi: 10.1186/s12874-024-02168-8 (PMC10924983; doi:10.1186/s12874-024-02168-8)
Supplement: Supplementary file 5 — Additional file 5. Individual assessment and consensus. [file 12874_2024_2168_MOESM5_ESM.pdf]

An assessment of the informative value of data sharing statements in clinical trial registries (Ohmann et al.)

Additional file 5

Individual assessment and consensus

Categories:

**unclear**	DSS not understood, and in particular the plans for sharing IPD are not clear or understood
**no sharing**	DSS states or implies that there will be no sharing of IPD. Different specific reasons may be provided.
**no plans**	DSS states that there are currently no plans regarding IPD sharing or a decision has not yet been made
**vague+**	DSS states or more normally implies that there will some degree of IPD sharing, but no details are given or sharing appears limited.
**request+**	DSS implies that IPD can be obtained by request, though who to may not be explicitly stated. If stated it is usually the investigator, sometimes the sponsor.
**storage+**	DSS states that IPD will be transferred to a repository – may be general, specific or institutional. More often not named specifically.
**complex+**	DSS states or implies that IPD will be available but under a more complex regime than any of those described above.
**unclear**	DSS not understood, and in particular the plans for sharing IPD are not clear or understood

srce = registry ID

srce Id	data sharing statement	expert B	expert A	consensus
ACTRN12620000447954	What data will be shared? Data on baseline characteristics, outcomes and drug levels, after de-identification When will data be available (start and end dates)? From 3 months following publication until 2 years after publication Available to whom? Only researchers who provide a methodologically sound proposal and who demonstrate capacity to analyse pharmacokinetic data Available for what types of analyses? Pharmacokinetic analysis By what mechanism will data be made available? Email to the Principal Investigator listed in this record	request+	request+	request+
ACTRN12620000512921	What data will be shared? All data collected can be shared in the context of relevant ethical and regulatory requirements When will data be available (start and end dates)? Data will be available once analysis has been completed for this study for a period of 5 years after analysis Available to whom? Researcher who request the data for a specific project that has IRB/ethics approval. Available for what types of analyses? Data can be used with permission of the investgators for any purposes provided it does not contravene ethics or regulatory requirements By what mechanism will data be made available? By contacting the investgators by email (shane.george@uq.edu.au)	complex+	request+	complex+
ACTRN12620000527965	What data will be shared? All of the individual participant data collected during the trial, after de-identification When will data be available (start and end dates)? Beginning 3 months following main results publication; no end date determined Available to whom? Researchers who provide a methodologically sound proposal Available for what types of analyses? To achieve the aims in the approved proposal By what mechanism will data be made available? Access subject to approvals by Principal Investigators Javeed.Travadi@health.nsw.gov.au j.oei@unsw.edu.au	request+	request+	request+

ACTRN12620000779976	What data will be shared? All of the individual participant data collected during the trial, after de-identification When will data be available (start and end dates)? Available following publication of the trial results. No end date determined Available to whom? Case-by-case basis at the discretion of Primary Sponsor Available for what types of analyses? Only to achieve the aims in the approved proposal determined on a case-by-case basis at the discretion of Primary Sponsor. By what mechanism will data be made available? Individuals may request data from the primary investigator, Sarah Egan, by emailing S.Egan@exchange.curtin.edu.au.	request+	request+	request+
ACTRN12621000420842	What data will be shared? Individual participant data that support any results reported in a published article after de-identification. When will data be available (start and end dates)? Beginning 3 months and ending 5 years following article publication. Available to whom? Data will be available for researchers when the use of this de-identifiable data has been approved by a reviewing committee of investigators and an independent ethics committee. Available for what types of analyses? Data analysis By what mechanism will data be made available? Data can be obtained from primary investigator (PI) via email: Gustavo.duque@unimelb.edu.au or Via AIMSS generic email and clinical trials officer (Petra Marusic: petra.marusic@unimelb.edu.au)	complex+	request+	complex+
ACTRN12621001254886	What data will be shared? Only deidentified individual participant data of published results will be available as per Participants Information Letter and Consent Forms When will data be available (start and end dates)? 1 July 2024 - 31 June 2025 Available to whom? Available to researchers upon an email request Available for what types of analyses? To be decided By what mechanism will data be made available? by emailing ewa.geba@acu.edu.au	request+	request+	request+

ACTRN12621001412820	What data will be shared? Identifiable data will not be publicly released, however deidentified individual participant data including relevant demographic and health information, primary outcome (neutralising antibody), and secondary outcomes (other measures of immunological response, measures of vaccine safety/tolerability, measures of study medication safety/tolerability) will be made available as per below criteria. When will data be available (start and end dates)? 3 months following publication of all study results. Data will no longer be available after 10 years. Available to whom? Applications from experienced investigators for trial data can be made through correspondence with the Principal Investigator (or the corresponding author in the case of published works) Available for what types of analyses? All reasonable requests from experienced investigators will be considered, including re-analysis of trial results, secondary outcomes analysis, and sub-group analysis By what mechanism will data be made available? After approval, deidentified data will be provided through access on a secure electronic platform. Approval can be sought through contacting the Principal Investigator by email (toby.coates@sa.gov.au), or the Corresponding Author for published data.	complex+	request+	request+
DRKS00021506	Informed consent is obtained in the local institutions. Forms are provided via the DGPI Homepage. The Data is saved in anonymous form on the DGPI Server. Only local Institutions can identify patients. Transfer of anonymous data between Centers or international databases is possible. Responsible for the data is the PI (Jakob Armann). Data will be saved for maximal 10 years after the study period has ended and the data is analyzed.	complex+	vague+	vague+
DRKS00022155	no plan for IPD sharing	no plans	no plans	no plans
DRKS00022387	Within the research network of the pta (Bertha von Suttner University and ÖAGG) the data will be processed jointly	unclear	unclear	unclear
DRKS00023180	Pseudonymized data can be made available on request.	request+	request+	request+
DRKS00024578	The study results are provided either as a supplement in anonymized form as an Excel table or on request.	request+	request+	request+
DRKS00024668	Data will not be shared due to monocentric observational character of this study.	no sharing	no sharing	no sharing
DRKS00024695	anonym data should be shared with peers for scientific validation.	vague+	vague+	vague+

DRKS00025497	The screening data, the preoperative, intraoperative and postoperative data of the patients will be made available in anonymised form with other study centers after application and approval of the corresponding application.	request+	request+	request+
DRKS00026184	The data will initially be used within the consortium. However, it is possible to pass on the data to external scientists after a suitable proposal has been positively evaluated by the consortium. In addition to biomaterials (blood, immune cells, urine, etc.), molecular and medical (PDMS) data will then be available.	vague+	complex+	complex+
DRKS00027370	Centers will receive your center-specific evaluation via the publication and via the study groups.	unclear	no sharing	unclear
DRKS00028689	Special laboratory analysis are not compareable to other laboratory analysis.	unclear	unclear	unclear
IRCT20090803002276N4	What will be shared: All data, after the individuals are unidentified, can be shared When: Six months after publishing the results To whom: Academic researchers Conditions: Scientific purposes Where to obtain: Website of the Research Committee of Qazvin University of Medical Sciences How to obtain: Clear request on the site to access the data by the individual and then review the request by the research assistant within 2 weeks and then allow access to the data. Comments:	request+	request+	request+
IRCT20091012002582N22	What will be shared: All potential data can be shared after making peoples unrecognizable. When: Starting 6 months after publication . To whom: Documents will be available for people working in academic institutions and also people working in businesses. Conditions: There will be no specific limitations to the utilization of the data Where to obtain: Dr .Ata Mahmoodpoor, Department of Anesthesiology, Faculty of Medicine, Golgasht Street, Tabriz East Azarbaijan Islamic Republic of Iran ,Phone+98 413 3341994, Fax+98 41 33341994 , amahmoodpoor@yahoo.com How to obtain: Applicants will access the data from the present study by sending an email to the responsible author for a maximum of one week. Comments:	request+	request+	request+

IRCT20100228003449N31	What will be shared: Results of the study will be published. The study protocol and statistical analysis will be included in the manuscript When: One year after finishing the study, data will be published and will be available in databases To whom: After permission form the sponsor, data of the study will be available for academic researchers, physicians and scientific institutes Conditions: Other researchers are permitted to included the results in their systematic reviews and metaanalysis Where to obtain: For this you may ask Hossein Khalili through following information: Department of Clinical Pharmacy, Faculty of Pharmacy, Tehran University of Medical Sciences, 16 Azar Ave.,Tehran, Iran Postal code: 1417614411 E-mail: khalilih@tums.ac.ir How to obtain: After receiving the query, dependent on the requested data, the scientific responsible person of the study will response to the query in coordinate with the sponsor within 2 weeks Comments:	request+	request+	request+
IRCT20111224008507N6	What will be shared: Part of the data is accessible When: starting in junuary 2022 To whom: Physicians Conditions: Systematic review articles Where to obtain: Contact Dr.Mohammad Sadegh Rezai. Email: drmsrezaii@yahoo.com How to obtain: Informations will send within few days after the call. Comments:	request+	vague+	vague+
IRCT20130812014333N164	What will be shared: The main outcomes of the study will be shared. When: 6 months To whom: If requested, results will be made available to other academic researchers Conditions: Collected data is confidential and will not be shared with anyone else Where to obtain: Send E-mail to the responsible for the update to get the documentation How to obtain: Documentation will be emailed within a 15-day timeframe Comments:	request+	no sharing	no sharing
IRCT20151227025726N28	What will be shared: All potential data can be shared after blinding When: Six months after publishing the results To whom: Researchers working in academic institutions Conditions: For research purposes and meta-analysis studies Where to obtain: Dr. farzaneh Dastan, Dr. Masih Daneshvari Hospital, Daar-Abad, Niavaran How to obtain: Official letter to the researchers through Email (fzh.dastan@gmail.com). Comments:	request+	request+	request+

IRCT20160118026097N3	What will be shared: All potential data can be shared after people have not been identified When: Start the access period 6 months after printing the results To whom: It will be available for researchers working in academic and scientific institutions Conditions: According to the rules of the COPE Where to obtain: jvafaeemanesh@yahoo.com How to obtain: Email the responsible author Comments: no	request+	vague+	vague+
IRCT20170211032494N3	What will be shared: All collected Individual Participant Data (IPD) will be shareable after being anonymized When: Document files will become available indefinitely 2 months after publication of the results To whom: Researchers of academic institutions will be given access to the required data. Conditions: All types of analysis are permitted. All acquired data will be shareable if the requester is identified as an academic researcher by the study supervisor. Where to obtain: Data will be accessible through Dr Nima Mousavi Darzikolaee; Email: Mousavi.nima@yahoo.com/ mousavi-n@razi.tums.ac.ir How to obtain: The applicants should send an email to the provided email addresses indicating their scientific and academic identity, and declare where they will be using this data and for what intention, and state that their published work will cite the relevant researchers. In this case they will be able to obtain the relevant data files. Comments:	request+	request+	request+

IRCT20180226038870N1	What will be shared: All data is potentially shareable after unidentified individuals When: Access starts 6 months after the results are published To whom: People working in academic and scientific institutions Conditions: The data can be used only with the permission of the author responsible for the study and only for scientific purposes such as sharing in scientific situations. In order to send a request for access to data or documents, it is necessary for the requesting person to send an e.mail to the responsible author and state the purpose of the request for access to data. Where to obtain: To receive data and documents related to the project, it is necessary to send an e.mail to the responsible author. Named: Mehrshad Poursaeed Esfahani e.mail: mpoorsaid@yahoo.com Address: Shahid Chamran Highway, Yemen St., Daneshjoo Blvd., Shahid Taleghani Hospital And contact number: 982122439948 How to obtain: After sending the e.mail to the responsible author, within 1-2 weeks, the responsible author will send the appropriate answer to the requesting person. Comments:	request+	request+	request+
IRCT20180712040449N2	What will be shared: The documentation including the primary and secondary outcomes of patients that will be available When: From the start of the clinical study to one year after the completion of the clinical study To whom: If another researcher wants to perform a clinical study in this field and needs our information to do the study, the information will be provided with confidentiality. - If patients have side effects, and their doctors need the treatment information. -If a health authority’s health policy requires our study information, then the information is provided with confidentiality. Conditions: If the applicant is authenticated, his request will be discussed with other researchers involved in the study, and the result will be informed to him. Where to obtain: Mehrdad Karimi mehrdadkarimi@yahoo.com 0098 21 66976527 How to obtain: At first, the applicants must send their request to the correspond author of the study, whose email address is on the IRCT site, and after verifying the applicant’s identity, including one of the upon items, the correspond author requests information of the researchers who are present in the study and is sent to applicants. This period, after the applicant’s authentication, will take about two weeks. Comments:	request+	request+	request+

IRCT20180728040617N3	What will be shared: Some of the patient and caregivers' demographic information can be shared after unidentifying individuals. All of the caregivers quality of life, caring burden and patients psychological distress assessment information can be shared with caregivers after unidentified individuals. When: After publishing the article To whom: Project partners Conditions: People who do similar research Where to obtain: It will be done by email with the person in charge of the project. How to obtain: Data is made available to individuals via email. Comments:	request+	request+	request+
IRCT20181030041504N1	What will be shared: Main outcome data, statistical analysis, final report, informed consent form When: 6 months after publish the results To whom: Academic researchers Conditions: Master of Medical Sciences PhD in Medical Sciences specialist Contribute to the advancement of science Where to obtain: Email m.mohamadpour2817@yahoo.com Contact number: 09120031782 Dr. Masoomeh Mohamadpour - Tehran How to obtain: 48-72 hours after sending the email request Comments:	request+	no sharing	no sharing
IRCT20190406043175N3	What will be shared: Dissemination of information after completing the clinical trial and publication of the article taken from the plan When: After publishing the article To whom: Researchers Conditions: For research reasons Where to obtain: Sending Email to the researchers How to obtain: Sending Email to the researchers Comments:	request+	request+	request+
IRCT20200217046526N1	What will be shared: All collected deidentified IPD can be shared When: 6 months after publication To whom: Researchers and clinicians Conditions: Planning of similar studies in other academic centers Where to obtain: Email How to obtain: 1-2 months after request Comments:	request+	request+	request+
IRCT20200328046886N1	What will be shared: all collected deidentified IPD When: starting 1 month after publication To whom: people working in academic institutions Conditions: in situation that invastigator willing Where to obtain: email addresses How to obtain: in short time after request with email will be available Comments:	request+	request+	request+

IRCT20200411047023N1	What will be shared: After issuing appropriate permission from Golestan University of Medical Sciences all recorded data can be shared anonymously. When: 6 months after publishing the results of the study in research journals. To whom: All the researchers and scientific institutes can access our data. Conditions: All the researchers and scientific institutes should mention why and how they want to use the data in their request. Moreover, distributing further results needs new permission from the corresponding person. Where to obtain: Written formal requests should be emailed to Dr. Najmeh Shahini using the below email address: Najmeh.shahini@gmail.com 5th Azar Hospital, 5th Azar St., Gorgan, Iran Phone Number: 00981731153365 How to obtain: After receiving formal data access requests, it should be evaluated and approved by the research team and deputy of research at Golestan University of Medical Sciences. Comments:	request+	request+	request+
IRCT20200412047042N1	What will be shared: The main outcomes of the study will be shared. When: One month after publishing To whom: If requested, results will be made available to other academic researchers Conditions: Collected data is confidential and will not be shared with anyone else Where to obtain: Send E-mail to the responsible for the update to get the documentation How to obtain: Documentation will be emailed within a 30-day timeframe Comments:	no sharing	no sharing	no sharing
IRCT20200418047119N1	What will be shared: The results of the study and the collected data will be published in the form of an article When: After the paper is published To whom: All clinical researchers Conditions: The data can only be used for review studies or to find a cure for an epidemic. Where to obtain: Dr Sajad Moradi from Kermanshah University of Medical Sciences, send request by sajadmoradi28@gmail.com How to obtain: The data will finally reach the applicant one week after the request Comments:	request+	vague+	vague+
IRCT20200515047456N1	What will be shared: Demographic information When: Six month after publication To whom: Researchers who work in university Conditions: Only for information Where to obtain: Via e.mail How to obtain: Send a request via e.mail Comments:	request+	request+	request+

IRCT20200624047908N1	What will be shared: All data can be shared after the participants in the study are unrecognizable. When: The data access period after publishing of the article To whom: The data in this study will be available for researchers working in academic and scientific institutions, as well as the Food and Drug Administration. Conditions: Any analysis can be done by main researcher permission. Where to obtain: gholamali.eslami1351@gmail.com How to obtain: The researcher or pharmaceutical company can send their request to the academic email of project manager. After ensuring the accuracy of the submitted documents, the project manager will provide the requested information to the researcher or pharmaceutical company in of one week. Comments:	request+	request+	request+
IRCT20200723048178N1	What will be shared: Primary outcome When: Data will be available for six months after its collection To whom: Healthcare workers Conditions: Data may be requested via email Where to obtain: individuals can ask for it via the personal email of the investigator How to obtain: Data may be available within a week after the request. Comments:	request+	request+	request+
IRCT20201019049079N1	What will be shared: Measured variables are available after unidentifiable individuals. When: Up to one year after publish results. To whom: All people in the research centers. Conditions: Allowed for any use. Where to obtain: Mohammad Amin Fallah, 09135258850, amin.fallah44@gmail.com How to obtain: After contacting and knowing the identity of the person, information will be provided to them. Comments:	request+	unclear	unclear
IRCT20201214049709N1	What will be shared: Deidentified IPD related to outcome will be shared. When: The access period will begin once the study is complete and the main results have been published in peer reviewed journals. To whom: The data that have been published in peer reviewed journals, will be available just for academic researchers. Conditions: The proposed study protocol should be submitted to RAZI vaccine and serum research institute and approved by its scientific and technical committee. Where to obtain: After publishing the article researchers can submit their request to Dr. Mohammad Hossein Fallah at the following email address (mhf2480@yahoo.com) How to obtain: Data will be made availabe after consideration and approval by the relevant authorities from Razi Vaccine and Serum Research Institute. Comments: -	complex+	request+	complex+

IRCT20210324050760N1	What will be shared: Only part of the data such as information about the main outcome or the like can be shared. When: 2021-2022 To whom: The data will be available only to researchers working in academic and scientific institutions Conditions: I have not decided yet Where to obtain: I have not decided yet How to obtain: I have not decided yet Comments:	vague+	vague+	vague+
IRCT20210427051100N1	What will be shared: All information can be shared two months after the results are published When: Two months after the publication of the results To whom: Physicians, nurses, and infectious disease specialists Conditions: To evaluate other complementary therapies and compare its effect with existing treatment related to COVID-19 disease Where to obtain: Send an email to mortezakhamoushi@gmail.com How to obtain: Two months after the results are published, send a written request to mortezakhamoushi@gmail.com. In this case, and finally within one month after receiving the email, the request will be answered. Comments:	request+	request+	request+
IRCT20210613051560N1	What will be shared: paper publication When: next one years To whom: all researchers Conditions: research use Where to obtain: Taraneh Naghibi How to obtain: By sending an email Comments:	unclear	unclear	unclear
ISRCTN11721294	IPD sharing statement: All data generated or analysed during this study will be included in the subsequent results publication.<br>IPD: Other	vague+	unclear	unclear
ISRCTN12107048	General dissemination plan: Planned publication in a high-impact peer-reviewed journal.<br>IPD: Available on request	request+	request+	request+
ISRCTN15317247	IPD sharing statement: The datasets generated during and/or analysed during the current study are not expected to be made available. NHSEI remains the data controller of the data. No plans for releasing the data have been discussed with NHSEI, and given the sensitive nature of the topic (COVID-19 vaccination rates), we expect NHSEI not to wish to make the data available.<br>IPD: Not expected to be available	no sharing	no sharing	no sharing

ISRCTN15674970	General dissemination plan: Results will be published, presented at conferences and made available for use by other researchers. The researchers will host a study-specific website and Twitter account and will share lay summaries coproduced with our patient partners.<br><br>Patient and public involvement<br>People with COVID lived experience have co-designed this research proposal. A lived experience advisory panel will work with the researchers throughout the project and meet regularly to contribute to all aspects of the study.<br>IPD: Available on request	request+	request+	request+
ISRCTN15689873	IPD sharing statement: The datasets generated during and/or analyzed during the current study are not expected to be made available as no ethical permission have been obtained to share the participant-level data outside the research group. The data will be held at secure servers at the Swedish School of Sport and Health Sciences.<br>IPD: Not expected to be available	no sharing	no sharing	no sharing
ISRCTN15779782	General dissemination plan: This international collaboration is coordinated through the World Health Organisation, which is also a sponsor of the trial. Any wholly reliable interim findings will be disseminated rapidly by the WHO. There will be group authorship recognizing the contribution of all national and local investigators and guided by the International Committee of Medical Journal Editors (ICMJE) recommendations. Alt-hough the writing committee will consist of the executive group and the WHO trial secretariat, authorship will include all steering committee members and local collaborators whose hospital, in the view of the national principal investigator, contributed substantially towards the trial.<br>IPD: Stored in repository	storage+	storage+	storage+

ISRCTN17782018	General dissemination plan: Study documents are not currently available but will be made available on request. Exact details of the publication policy are still being developed. However, the researchers will provide rapid recommendations to drive tailored public health initiatives for cancer early detection and prevention in the pandemic recovery period. Longer-term, the study will lay the foundations for assessing policy impact in response to future global health threats.<br>IPD: Data sharing statement to be made available at a later date	no plans	no plans	no plans
ISRCTN17834262	General dissemination plan: Submitted for publication in Plos One<br>IPD: Data sharing statement to be made available at a later date	no plans	vague+	no plans
ISRCTN18015802	General dissemination plan: Planned publication in a high-impact peer-reviewed journal.<br>IPD: Stored in non-publicly available repository	storage+	storage+	storage+
ISRCTN32891583	IPD sharing statement: The data-sharing plans for the current study are unknown and will be made available at a later date.<br>IPD: To be made available at a later date	vague+	vague+	vague+
ISRCTN38746119	General dissemination plan: Planned publication in:<br>- High-impact peer-reviewed journals<br>- Abstracts submission to national and international conferences<br>- Social media including Twitter, YouTube, LinkedIn<br>IPD: Data sharing statement to be made available at a later date	no plans	no plans	no plans
ISRCTN48734830	General dissemination plan: Planned publication in peer-reviewed journal.<br>IPD: Not expected to be made available	no sharing	no sharing	no sharing
ISRCTN54053617	General dissemination plan: The impact of this work may be significant in understanding and eventually managing patients with COVID 19 and in other enveloped viruses. The data, once analysed, will initially be disseminated via publication in peer-reviewed scientific journals with minimum delay.<br>IPD: Available on request, Other	request+	request+	request+

ISRCTN56609224	IPD sharing statement: Non-identifiable datasets generated during and/or analysed during the current study will be available for academic use upon request from PHE's Office for Data Release (ODR) https://www.gov.uk/government/publications/accessing-public-health-england-data/about-the-phe-odr-and-accessing-data, subject to their proposed use being compatible with the consent obtained from individuals, and an analysis of the risk of identifying individuals.<br>IPD: Available on request	request+	request+	request+
ISRCTN69254139	General dissemination plan: Publication policy:<br>The Investigators will be involved in reviewing drafts of the manuscripts, abstracts, press releases, and any other publications arising from the study. Data from the study may also be used as part of a thesis for a PhD or MD.<br>IPD: Data sharing statement to be made available at a later date	no plans	no plans	no plans
ISRCTN89159899	General dissemination plan: Planned publication in a high-impact peer-reviewed journal.<br>IPD: Other	unclear	unclear	unclear
ISRCTN99248812	General dissemination plan: 1. The full study protocol is not yet available but will be uploaded to the registration prior to study completion.<br>2. Planned publication of the study results in a high-impact peer-reviewed journal.<br>IPD: Data sharing statement to be made available at a later date	no plans	no plans	no plans
JPRN-jRCTs031200196	The clinical data obtained in this study will be secondarily used after the report of the study is completed (in the condition that data anonymization and privacy issues are protected). Especially, the comparison in effect between the newly approved drugs in Japan (eg., Remdesivir, Baricitinib) vs. mono therapy or combination therapy in this study will be retrospectively conducted.	vague+	vague+	vague+
KCT0006152	: garymh@kcch.re.kr	request+	request+	request+

NCT04247620	(As of June 2022): Participants receive informed consent form, and other participant data is not returned to participant.<br>Time frame: Receive copy of signed informed consent form upon signing<br>Access Criteria: All participants receive copy of signed informed consent form<br>Information available: Informed Consent Form (ICF)	unclear	unclear	unclear
NCT04320056	(As of April 2020): No There is not plan to share individual participant data. All data if shared with be de-identified. Data will be stored on a secure server with access only by study personal.	no plans	vague+	no plans
NCT04329832	(As of April 2022): In order to protect patient privacy and comply with relevant regulations, identified data are unavailable. Requests for deidentified data from qualified researchers with appropriate ethics board approvals and relevant data use agreements will be processed by the Intermountain Office of Research, officeofresearch@imail.org.	complex+	request+	complex+
NCT04330261	(As of May 2022): In keeping with the joint statement on sharing research data and findings relevant to the novel coronavirus (nCoV) outbreak, this study will share data rapidly with local governments as well as international stakeholders.<br>Time frame: Ideally in real-time, over the next 18 months<br>Information available: Study Protocol, Statistical Analysis Plan (SAP)	vague+	vague+	vague+
NCT04336410	(As of April 2022): Data dictionaries and all collected IPD will be stripped of identifiers and may be made available upon request.<br>Time frame: Anonymous IPD may be shared following or during the publication of summary data. Archival data may be accessed for up to 10 years following the end of the study.<br>Access Criteria: Those who request the anonymous IPD must provide a plan of study explaining how the data will be used. Requests may be sent to the Central Contact Person. Requests will be reviewed based on the potential for the planned use of the IPD for advancing scientific knowledge and theory.<br>Information available: Study Protocol, Informed Consent Form (ICF)	request+	complex+	complex+
NCT04338009	(As of April 2021): Not making it available	no sharing	no sharing	no sharing
NCT04341675	(As of May 2020): De-identified aggregate data will be made available to other researchers on a case-by-case basis.	vague+	vague+	vague+

NCT04343001	(As of April 2020): As many sites will contribute to this trial, individual sites cannot restrict the publication of the manuscript relating to the outcomes of this trial. All anonymised data from this trial will be made freely available on our data sharing site: http://freebird.lshtm.ac.uk.<br>Time frame: Within 6 months or sooner of publication<br>Access Criteria: Log-in required for the sole purpose to monitor data download.<br>URL: http://freebird.lshtm.ac.uk<br>Information available: Study Protocol, Statistical Analysis Plan (SAP), Informed Consent Form (ICF)	storage+	storage+	storage+
NCT04343976	(As of October 2021): Coded data is anticipated to be shared with outside institutions. Safety data will be shared with the drug manufacturer<br>Time frame: Data is anticiapted to be made available within 3 months of study completion. Safety data, specifically treatment related adverse events, will be shared in real time<br>Access Criteria: researchers accessing IPD must be an approved and have a data use agreement in place with Partners Healthcare to access the data<br>Information available: Study Protocol, Informed Consent Form (ICF)	complex+	complex+	complex+
NCT04346082	(As of July 2021): may share data on request of investigators	request+	request+	request+
NCT04349410	(As of October 2020): Data will be made available through electronic request from approved individuals and institutions.<br>Time frame: This will depend upon the availability of staff given the multi-nation approach to this project.<br>Access Criteria: Expressed request through email as listed.<br>Information available: Study Protocol, Statistical Analysis Plan (SAP), Informed Consent Form (ICF), Clinical Study Report (CSR), Analytic Code	request+	request+	request+
NCT04355442	(As of April 2020): NC	unclear	no plans	unclear

NCT04355767	(As of October 2021): The complete de-identified patient data set will be shared.<br>Time frame: Data will be available indefinitely.<br>Access Criteria: Data requests will be managed by National Heart, Lung, and Blood Institute (NHLBI).<br>URL: https://biodatacatalyst.nhlbi.nih.gov/<br>Information available: Study Protocol, Statistical Analysis Plan (SAP), Informed Consent Form (ICF)	complex+	request+	complex+
NCT04356534	(As of July 2020): Monitoring, audits, and REC review will be permitted and provide direct access to source data and documents. The Lead PI and the researchers assigned by him will have access to the stored data/specimens. Only the Lead PI and the researchers assigned working on this study will be eligible to obtain the data/specimens from the participants during data collection.<br>Time frame: Dr Manaf will act as the data custodian and is responsible for the storage, handling and quality of the study data.<br><br>Data will be collected in the case report form to allow for cross referencing to check validity.<br><br>Study documents (paper and electronic) will be retained in a secure (kept locked when not in use) location during and after the trial has finished. All essential documents including source documents will be retained for a period of 5 years after study completion (last patient, last study point). A label stating the date after which the documents can be destroyed will be placed on the inside front cover of the case notes of trial participants.<br>Access Criteria: Study documents (paper and electronic) will be retained in a secure (kept locked when not in use) location during and after the trial has finished.<br>Information available: Study Protocol, Statistical Analysis Plan (SAP), Clinical Study Report (CSR), Analytic Code	unclear	no sharing	no sharing

NCT04357327	(As of November 2020): We could provide anamnestic, clinical and serological data of each participant at the end of the study, or at least at the end of the first phase.<br>Time frame: Data will be available at the end of the study, once statistical analyses is conducted<br>Access Criteria: Contact Principal Investigator<br>Information available: Study Protocol, Statistical Analysis Plan (SAP), Analytic Code	request+	request+	request+
NCT04359797	(As of December 2021): Individual participant data that underlie the results reported will be made available (including data dictionaries) after de-identification.<br>Time frame: The data will become available 3 months following publication of outcomes and will remain available for at least 5 years.<br>Access Criteria: Data will be made available to researchers who provide a methodologically sound proposal that has been approved by the Vanderbilt Institutional Review Board and the study executive committee.<br>Information available: Study Protocol, Statistical Analysis Plan (SAP), Analytic Code	complex+	complex+	complex+
NCT04364594	(As of July 2020): Study Protocol approved by Ethical Committee, informed consent, clinical study report, will be available<br>Time frame: The data will be available immediately after the statistical analysis of the results and will remain available for at least 1 year<br>Access Criteria: On request by e-mail<br>Information available: Study Protocol, Informed Consent Form (ICF), Clinical Study Report (CSR)	request+	request+	request+
NCT04367363	(As of December 2020): Due to stipulations by the Institutional Review Board, data cannot be shared.	no sharing	no sharing	no sharing
NCT04368884	(As of July 2020): We have not yet prepared an IPD sharing protocol at this stage. Data might be shared if requested.	no plans	vague+	no plans

NCT04374071	(As of May 2020): Data will be shared One year from publication<br>Time frame: One year from publication<br>Access Criteria: All requests for IPD should be referred to the PI by email.<br>Information available: Study Protocol, Statistical Analysis Plan (SAP), Clinical Study Report (CSR), Analytic Code	request+	request+	request+
NCT04374617	(As of May 2020): We have planned to write a paper	vague+	no sharing	no sharing
NCT04377100	(As of April 11, 2022): .We will likely submit the de-identified data to a repository but do not have clear plans yet. We will update as we determine the plans.	no plans	no plans	no plans
NCT04382053	(As of April 2022): Novartis is committed to sharing with qualified external researchers, access to patient-level data and supporting clinical documents from eligible studies. These requests are reviewed and approved by an independent review panel on the basis of scientific merit. All data provided is anonymized to respect the privacy of patients who have participated in the trial in line with applicable laws and regulations.<br><br>This trial data availability is according to the criteria and process described on www.clinicalstudydatarequest.com.	complex+	storage+	storage+
NCT04382495	(As of March 2022): email	request+	request+	request+

NCT04387968	(As of October 2021): Blood sample to be taken after self-bite. A consent form is given to the officer.<br><br>A drop of blood is deposited on a coverslip, 2 drops of reagents allow migration to the gel.<br><br>Reading in 15 minutes<br><br>strip: negative result<br>bands: positive result<br><br>An authorized person, subject to medical confidentiality, reports the result in the medical file and informs the agent of the result.<br><br>He is given a paper document signed by the SMP with the procedure to follow.	unclear	unclear	unclear
NCT04388514	(As of May 2020): all IPD that underlie results in a publication<br>Time frame: starting 6 months after publication<br>Access Criteria: Contacting the Principal Investigator<br>Information available: Study Protocol, Statistical Analysis Plan (SAP), Clinical Study Report (CSR)	request+	request+	request+
NCT04388683	(As of April 2022): Data from this pilot study will be used to plan future a larger randomized controlled outcome trial.	unclear	unclear	unclear

NCT04398264	(As of April 2022): Since COVID-19 in pregnant women is still a novel topic, we anticipate the need for collaboration with other researchers studying the effects of COVID-19 in pregnant women.<br><br>Individual participant data that underlie the results to be reported in our research would be available to be shared after deidentification (text, tables, figures and appendices) for individual participant meta-analysis.<br><br>Proposals may be submitted up to 36 months following article publication. After 36 months the data will be available in our Institution's data warehouse but without investigator support other than deposited metadata.<br><br>Time Frame: Beginning 9 months and ending 36 months following article publication.<br><br>Information to be shared: Study Protocol, Results.<br><br>Access Criteria: Investigators whose proposed use of the data has been approved by an independent review committee identified for this purpose.	storage+	complex+	complex+
NCT04403477	(As of May 2020): Data will be shared with the journal authority and make public as part of the publication<br>Time frame: six months<br>Access Criteria: Available on public domain like figshare, researchgate and others<br>Information available: Statistical Analysis Plan (SAP), Informed Consent Form (ICF), Clinical Study Report (CSR)	vague+	storage+	vague+
NCT04405310	(As of December 2020): IPD will be shared by petition.<br>Time frame: 3 months after completion up to five years<br>Access Criteria: proposal should be directed to gmemiinv@gmails.com, to gain access, requestor will need to sign a data access agreement<br>Information available: Study Protocol, Statistical Analysis Plan (SAP), Informed Consent Form (ICF), Clinical Study Report (CSR)	request+	request+	request+

NCT04405934	(As of February 2022): The fully anonymised datasets analysed during the study will be stored on a publicly available repository. The COG-UK HOCI study to be shared on the UCL Data Repository data-sharing platform so that the data may be reused by other researchers. The protocol will also be shared.<br>Time frame: This will be done with 6 months of public reporting of results, with data available for 5 years.<br>Access Criteria: Fully open access<br>Information available: Study Protocol	storage+	storage+	storage+
NCT04408157	(As of September 2021): Data will be confidentially and securely stored for 7 years as per University policy. Anonymised aggregated data will be made available upon request to the corresponding author.	request+	request+	request+
NCT04408456	(As of August 2020): Data will be shared with the concerned authority and Institutional Ethics committee as and when required.	vague+	no sharing	no sharing
NCT04410471	(As of May 2020): Multicenter study	unclear	unclear	unclear
NCT04413747	(As of August 2020): The datasets generated during and/or analysed during the current study will be available upon request within a web_ client structure<br>Time frame: 18 months<br>Access Criteria: ID (nikname) and password<br>URL: https://ifc.cnr.it<br>Information available: Study Protocol, Statistical Analysis Plan (SAP), Informed Consent Form (ICF), Clinical Study Report (CSR), Analytic Code	request+	vague+	vague+
NCT04416048	(As of December 2021): after protection period	vague+	vague+	vague+
NCT04433000	(As of June 2020): The whole anonymized dataset could be shared<br>Time frame: from the month of July, for one year<br>Access Criteria: on request by email<br>Information available: Study Protocol	request+	request+	request+
NCT04433039	(As of June 2020): The investigators will recommend further large group future researches for some notices in the study, but preferably not be biased	vague+	unclear	unclear

NCT04438850	(As of June 2021): The anonymized database will be uploaded into a public repository<br>Time frame: The database will be available upon publication of the results<br>Access Criteria: The data will be available in a public repository<br>Information available: Study Protocol	storage+	storage+	storage+
NCT04445428	(As of March 2022): Provided request, data can be made available	request+	request+	request+
NCT04446065	(As of September 2020): The results of this study will be published as open access article and all IPD that underlie results in a publication will be shared, preferentially as supplementary data set or available in a public repository. Any sensitive personal data/information will be not included.<br>Information available: Study Protocol, Statistical Analysis Plan (SAP)	storage+	vague+	storage+
NCT04448756	(As of May 2022): We are committed to enhancing public health through responsible sharing of clinical trial data. Following approval of a new product or a new indication for an approved product in both the US and European Union, the study sponsor and/or its affiliated companies will share study protocols, anonymized patient data and study level data, and redacted clinical study reports with qualified scientific and medical researchers, upon request, as necessary for conducting legitimate research. Further information on how to request data can be found on our website bit.ly/IPD21<br>Time frame: Within six months after the approval of a new product or a new indication for an approved product in both the United States and the European Union<br>Access Criteria: Qualified scientific and medical researchers can request the data. Such requests must be submitted in writing to the company's portal and will be internally reviewed regarding criteria for researchers' qualification and legitimacy of the research proposal.<br>URL: http://bit.ly/IPD21<br>Information available: Study Protocol, Statistical Analysis Plan (SAP), Clinical Study Report (CSR), Analytic Code	request+	complex+	complex+
NCT04453475	(As of June 2022): Individual participant data (IPD) will not be published. Other researchers are welcome to get in contact with the PI to get access to anonymous data.	request+	request+	request+
NCT04456426	(As of June 2020): Not by the moment	no plans	no plans	no plans

NCT04470583	(As of October 2020): Research team wishes to enable any meta-analyses of COVID-19 trials making appropriate requests. No plan to share IPD has been made at this time.	request+	request+	request+
NCT04473053	(As of September 2021): The research team actively encourage data sharing to expedite the development of COVID-19 treatments. The study team will provide data to other researchers on request and following the agreement of a data sharing plan. No confidential information regarding participants will be shared.<br>Time frame: 90 days after data analysis has been completed for each arm. This data will be available on request and a time frame can be discussed further when required.<br>Information available: Study Protocol, Statistical Analysis Plan (SAP), Informed Consent Form (ICF)	request+	request+	request+
NCT04484207	(As of October 2021): Data will be shared as needed with investigators, IRB etc. A plan will be determined in the short future<br>Time frame: two months<br>Access Criteria: Co-Is<br>Information available: Study Protocol, Statistical Analysis Plan (SAP), Informed Consent Form (ICF)	vague+	unclear	unclear
NCT04486144	(As of April 2021): KDunn and Associates, PA will de-identify data and archive it at The Schull Institute Data Archive at the University of Texas School of Biomedical Informatics after the Summer Research Program Conclusion.<br>Time frame: October 2020<br>Access Criteria: Need to have a specific question or plan to study further.<br>URL: http://www.theschullinstitute.org<br>Information available: Study Protocol, Statistical Analysis Plan (SAP), Clinical Study Report (CSR)	storage+	storage+	storage+

NCT04490473	(As of December 2020): It is planned to be published in the journal after the study is completed. The study will be shared with other researchers after it is published in the journal.<br>Time frame: will be shared after the work is finished.<br>Access Criteria: It will be shared with researchers who want to use the anxiety scale.<br>Information available: Study Protocol	vague+	vague+	vague+
NCT04493294	(As of July 2020): There are over 40 people involved in the study, we need their approval before data sharing	no plans	no plans	no plans
NCT04499677	(As of December 2021): No plan to share IPD has been made at this time.	no plans	no plans	no plans
NCT04508985	(As of August 2020): Data will be de-identified and shared among all collaborators.<br>Time frame: Immediate access to the data<br>Information available: Study Protocol, Statistical Analysis Plan (SAP), Informed Consent Form (ICF), Clinical Study Report (CSR)	vague+	unclear	unclear
NCT04512079	(As of January 2022): Individual participant data that underlie the results reported in this article, after de-identification (text, tables, figures, and appendices).<br>Time frame: Beginning 9 months and ending 36 months following article publication.<br>Access Criteria: Investigators whose proposed use of the data has been approved by an independent review committee ("learned intermediary") identified for this purpose. The type of analysis that will be conducted is for individual participant data meta-analysis. Proposals may be submitted up to 36 months following article publication. After 36 months the data will be available in our University's data warehouse but without investigator support other than deposited metadata.<br>Information available: Study Protocol	complex+	complex+	complex+
NCT04513184	(As of November 2020): Information exchange for research purposes<br>Time frame: Upon study completion, by request<br>Information available: Study Protocol, Clinical Study Report (CSR)	request+	request+	request+

NCT04525742	(As of October 2020): Study protocol was established by Berrin Huner and Ebru Yilmaz Yalcınkaya Statistical analysis (Power analysis) was established by Ozden Ozyemisci Taskiran Informed consent was written by Berrin Huner<br>Time frame: Research is planned to bu conducted between June 15 th and July 15 th 2020. After 15 th of July in 1 week coming statistical analysis will be finished and than in 3 weeks article will be written<br>Access Criteria: In necessary cases colleced data from the research will be shared with the local ethical committe<br>Information available: Study Protocol, Statistical Analysis Plan (SAP), Informed Consent Form (ICF)	unclear	no sharing	no sharing
NCT04527133	(As of August 2020): not planned	no sharing	no plans	no sharing
NCT04529408	(As of September 2021): Held within Imperial College London as per standard local data protection guidelines. Data may be considered for sharing upon reasonable request.	request+	request+	request+

NCT04564040	(As of December 2020): Qualified researchers can request access to anonymized individual patient-level data from<br><br>AstraZeneca group of companies sponsored clinical trials via the request portal. All requests will be evaluated as per the AZ disclosure commitment:<br><br>https://astrazenecagrouptrials.pharmacm.com/ST/Submission/Disclosure.<br><br>Yes, indicates that AZ are accepting requests for IPD, but this does not mean all requests will be shared.<br>Time frame: AstraZeneca will meet or exceed data availability as per the commitments made to the EFPIA Pharma Data Sharing Principles. For details of our timelines, please rerefer to our disclosure commitment at:<br><br>https://astrazenecagrouptrials.pharmacm.com/ST/Submission/Disclosure<br>Access Criteria: When a request has been approved AstraZeneca will provide access to the de-identified individual patient-level data in an approved sponsored tool. Signed Data Sharing Agreement (non-negotiable contract for data accessors) must be in place before accessing requested information. Additionally, all users will need to accept the terms and conditions of the SAS MSE to gain access. For additional details, please review the Disclosure Statements at:<br><br>https://astrazenecagrouptrials.pharmacm.com/ST/Submission/Disclosure<br>URL: https://astrazenecagroup-dt.pharmacm.com/DT/Home	complex+	complex+	complex+

NCT04568031	(As of November 2021): Qualified researchers can request access to anonymized individual patient-level data from AstraZeneca group of companies sponsored clinical trials via the request portal. All request will be evaluated as per the AZ disclosure commitment:<br>https://astrazenecagrouptrials.pharmacm.com/ST/Submission/Disclosure<br>Time frame: AstraZeneca will meet or exceed data availability as per the commitments made to the EFPIA Pharma Data Sharing Principles. For details of our timelines, please rerefer to our disclosure commitment at<br>https://astrazenecagrouptrials.pharmacm.com/ST/Submission/Disclosure<br>Access Criteria: When a request has been approved AstraZeneca will provide access to the de-identified individual patient-level data in an approved sponsored tool . Signed Data Sharing Agreement (non-negotiable contract for data accessors) must be in place before accessing requested information. Additionally, all users will need to accept the terms and conditions of the SAS MSE to gain access. For additional details, please review the Disclosure Statements at<br>https://astrazenecagrouptrials.pharmacm.com/ST/Submission/Disclosure<br>URL: https://astrazenecagroup-dt.pharmacm.com/DT/Home<br>Information available: Study Protocol, Statistical Analysis Plan (SAP)	complex+	complex+	complex+
NCT04581954	(As of September 2021): To be determined	no plans	no plans	no plans
NCT04592354	(As of October 2020): The plan is to publish the data so that it is available to other researchers.<br>Time frame: Data will become available after the conclusion of the study<br>Access Criteria: Unknown at this point.<br>Information available: Study Protocol, Informed Consent Form (ICF), Clinical Study Report (CSR)	vague+	request+	vague+
NCT04602871	(As of October 2020): No IPDs are to be shared with other researchers.	no sharing	no sharing	no sharing

NCT04638842	(As of October 2021): The information will be available in a private server or in a server of the journal(s) that we will publish the articles that will be the result of this study. The protocol of the study is currently in progress to be published, in this article will be included such study protocol, the informed consent is already shared in the register of clinical trials.<br>Time frame: This data will be available approximately in April 2021 and it will be permanently available. It will be shared in the databases of the journal where the article(s) will be published.<br>Access Criteria: Through the servers of the journal(s) where we will publish the articles.<br>Information available: Study Protocol, Informed Consent Form (ICF)	vague+	vague+	vague+
NCT04644172	(As of November 2021): This is pilot study. A major purpose of this study is simply to develop the intervention. Sharing individual data is premature.	no sharing	no sharing	no sharing
NCT04649944	(As of December 2020): IPD are not planned to be available	no sharing	no sharing	no sharing
NCT04649957	(As of May 2022): Not part of the study teams plans.	no sharing	no sharing	no sharing
NCT04654442	(As of September 2021): Grapheal and Grenoble University Hospital will decide to share IPD if possible according to GPDR regulation.	no plans	no plans	no plans
NCT04664075	(As of March 2022): The expectation is that after analysis the data from this study will be widely distributed in the medical and scientific community. Facilitated with presentations at local, national and international meetings, the hope is to publish widely in the medical literature. In addition there is an excellent media department at Imperial College that will publicise research that has public interest when it is published. All data will be anonymised and aggregated or pseudonymised; no identifying participant information will be published.<br>Time frame: Data will become available approximately 12 months from the last patient's last visit and remain available indefinitely<br>Access Criteria: According to study protocol<br>Information available: Study Protocol, Clinical Study Report (CSR)	vague+	vague+	vague+

NCT04697927	(As of October 2021): Memorial Sloan Kettering Cancer Center supports the international committee of medical journal editors (ICMJE) and the ethical obligation of responsible sharing of data from clinical trials. The protocol summary, a statistical summary, and informed consent form will be made available on clinicaltrials.gov when required as a condition of Federal awards, other agreements supporting the research and/or as otherwise required. Requests for deidentified individual participant data can be made beginning 12 months after publication and for up to 36 months post publication. Deidentified individual participant data reported in the manuscript will be shared under the terms of a Data Use Agreement and may only be used for approved proposals. Requests may be made to: crdatashare@mskcc.org.	request+	complex+	request+
NCT04705766	(As of April 2022): Research records will be kept confidential to the extent permitted by law. Subjects will be identified by a code, and personal information from study records will not be released without the subject's permission. Study subjects will not be identified in any publication that may result from this study. However, the records may be reviewed under guidelines of the Federal Privacy Act by site monitors to assure the accuracy and completeness of study data. The investigators will make sure patient health information is removed from all the bio-samples and data obtained. The investigators will adhere to the NIH and HHS policies regarding the sharing of data and resources with the scientific community, publications, and intellectual property rights, and sharing of biomedical research resources.	unclear	no sharing	unclear

NCT04713111	(As of January 2021): Under the 4YouandMe open source model, we will make all data, findings, digital health applications and algorithms available in the public domain. Accordingly, de-identified data produced from this project will be shared broadly with qualified researchers (among participants who opt in) through Sage Bionetworks Synapse and will serve as an immense resource, reflecting a highly granular and high-dimensional map of physiological stress responses and knowledge surrounding inadvertent consequences of objective stress measurement. Only data from consenting participants will be shared through Sage Bionetworks Synapse. Additionally, Source code for the developed app will be made available as open source software on GitHub so it can be evolved for future work by others.<br>Time frame: Internal collaborative researchers will have access to all coded data during the full duration of the study. Consented participants' coded data will be available in the Synapse at Sage Bionetworks for selected researchers to access indefinitely, one year after study completion.<br>Access Criteria: As coded study data will then exist among consenting participants in the Synapse at Sage Bionetworks in de-identified form, the electronic data files will be kept indefinitely. The Principal Investigator will be responsible for receipt and/or transmission of data as required.	storage+	vague+	storage+
NCT04730323	(As of January 2021): IPD can be shared with researchers in future too<br>Time frame: Whenever will be asked to, within few days<br>Access Criteria: Only officials can access the data<br>Information available: Study Protocol, Statistical Analysis Plan (SAP), Informed Consent Form (ICF), Clinical Study Report (CSR)	vague+	request+	vague+

NCT04743011	(As of September 2021): There is a plan to make IPD and related data dictionaries available<br>Time frame: The summary data will be published or made available 6 months after publication.<br>Access Criteria: Epidemiological data, clinical data, and patient evolution will be shared during the study only for researchers who request access to the data. Access requests will be analyzed by the main researcher, and they will only be released for scientific purposes.<br>Information available: Study Protocol, Statistical Analysis Plan (SAP), Informed Consent Form (ICF), Clinical Study Report (CSR)	request+	request+	request+
NCT04746443	(As of February 2021): The Data cannot be shared without the prior approval of the University	vague+	no sharing	unclear
NCT04760184	(As of February 2022): Upon request the pseudonymized data sheet will be made available for other researchers depending on purpose of the request.	request+	request+	request+
NCT04769284	(As of February 2021): The study design could be reported	unclear	no sharing	unclear
NCT04798027	(As of May 2022): Qualified researchers may request access to patient level data and related study documents including the clinical study report, study protocol with any amendments, blank case report form, statistical analysis plan, and dataset specifications. Patient level data will be anonymized and study documents will be redacted to protect the privacy of trial participants. Further details on Sanofi’s data sharing criteria, eligible studies, and process for requesting access can be found at: https://vivli.org	storage+	storage+	storage+
NCT04826822	(As of March 2021): Sharing IPD would require material transfer agreement according to the local rules and regulations. The study protocol, statistical analysis plan, and the translated version of the informed consent form can be shared upon request.	vague+	vague+	vague+
NCT04833010	(As of March 2022): Considered upon request.	request+	request+	request+
NCT04842435	(As of May 2021): All of the individual participant data collected during the trial, after deidentification<br>Time frame: Immediately following publication. No end date<br>Access Criteria: Anyone who wishes to access the data<br>Information available: Study Protocol, Statistical Analysis Plan (SAP), Informed Consent Form (ICF), Clinical Study Report (CSR), Analytic Code	vague+	vague+	vague+

NCT04845971	(As of April 2021): This is a multicenter trial. However, we reserve the possibility of enrolling further COVID-19 Hospital Units that are interested in participating in the trial, in order to get the correct sample size as needed for the study.Thus, if needed, we'll share IPD with the involved research centers.	vague+	unclear	no sharing
NCT04852978	(As of February 2022): All Individual Patient Data (IPD) that underlie publicly available results will be considered for sharing<br>Time frame: When Regeneron has received marketing authorization from major health authorities (e.g., FDA, European Medicines Agency (EMA), Pharmaceuticals and Medical Devices Agency (PMDA), etc.) for the product and indication, has made the study results publicly available (e.g., scientific publication, scientific conference, clinical trial registry), has the legal authority to share the data, and has ensured the ability to protect participant privacy.<br>Access Criteria: Qualified researchers can submit a proposal for access to individual patient or aggregate level data from a Regeneron-sponsored clinical trial through Vivli. Regeneron's Independent Research Request Evaluation Criteria can be found at: https://www.regeneron.com/sites/default/files/Regeneron-External-Data-Sharing-Policy-and-Independent-Research-Request-Evaluation-Criteria.pdf<br>URL: https://vivli.org/<br>Information available: Study Protocol, Statistical Analysis Plan (SAP), Informed Consent Form (ICF), Clinical Study Report (CSR), Analytic Code	storage+	storage+	storage+
NCT04879251	(As of May 2021): Will be discussed.	no plans	no plans	no plans

NCT04896541	(As of March 2022): Qualified researchers can request access to anonymized individual patient-level data from AstraZeneca group of companies sponsored clinical trials via the request portal. All request will be evaluated as per the AZ disclosure commitment: https://astrazenecagrouptrials.pharmacm.com/ST/Submission/Disclosure.<br><br>Yes, indeed that AZ are accepting requests for IPD, but this does not mean all requests will be shared.<br>Time frame: AstraZeneca will meet or exceed data availability as per the commitments made to the EFPIA Pharma Data Sharing Principles. For details of our timelines, please rerefer to our disclosure commitment at https://astrazenecagrouptrials.pharmacm.com/ST/Submission/Disclosure.<br>Access Criteria: When a request has been approved AstraZeneca will provide access to the de-identified individual patient-level data in an approved sponsored tool . Signed Data Sharing Agreement (non-negotiable contract for data accessors) must be in place before accessing requested information. Additionally, all users will need to accept the terms and conditions of the SAS MSE to gain access. For additional details, please review the Disclosure Statements at https://astrazenecagrouptrials.pharmacm.com/ST/Submission/Disclosure.<br>URL: https://astrazenecagroup-dt.pharmacm.com/DT/Home<br>Information available: Study Protocol, Statistical Analysis Plan (SAP)	complex+	complex+	complex+
NCT04898205	(As of May 2021): Upon conclusion of the study, we will share de-identified data upon request.	request+	request+	request+
NCT04910295	(As of May 2022): Data sharing of IPD will be discussed and decided upon request.	request+	request+	request+

NCT04931004	(As of June 2021): Individual participant data that underlie the results reported in this article, after deidentification (text, tables, figures, and appendices)<br>Time frame: Beginning 3 months and ending 5 years following article publication<br>Access Criteria: Researchers who provide a methodologically sound proposal<br>Information available: Study Protocol, Statistical Analysis Plan (SAP), Analytic Code	vague+	complex+	complex+
NCT04939428	(As of June 2022):<br>http://engagezone.msd.com/doc/ProcedureAccessClinicalTrialData.pdf<br>URL: http://engagezone.msd.com/ds_documentation.php	complex+	complex+	complex+
NCT04957017	(As of July 2021): There is not plan.	no plans	no plans	no plans
NCT04957082	(As of December 2021): Data will be made available per requirements and sharing structures established by NCI "SeroNet" initiative.<br>Time frame: Upon Study Completion<br>Information available: Analytic Code	complex+	vague+	vague+
NCT04969263	(As of October 2021): The plan is to share data upon completion of the study in: Immunology Database and Analysis Portal (ImmPort), a long-term archive of clinical and mechanistic data from DAIT-funded grants and contracts.<br>Time frame: On average, within 24 months after database lock for the trial.<br>Access Criteria: Open access.<br>URL: https://www.immport.org/home	storage+	storage+	storage+
NCT04982042	(As of July 2021): Clinical and demographic data of patients involved in this study will be made available to other researchers as requested.<br>Time frame: After 02 years<br>Access Criteria: The clinical and demographic data of the patients involved in this study will be made available to other researchers as requested after the end of the study and for an indefinite period.<br>URL: https://www4.unievangelica.edu.br/ppg/movimento-humano-e-reabilitacao/laboratorios<br>Information available: Study Protocol, Statistical Analysis Plan (SAP), Informed Consent Form (ICF), Clinical Study Report (CSR)	request+	vague+	vague+

NCT04987853	(As of July 2021): all IPD that underlie results in a publication<br>Time frame: starting 6 months after publication<br>Access Criteria: all types of supporting information will be shared with the interested party within the framework of the legislation of the Republic of Kazakhstan<br>URL: http://heartcenter.kz<br>Information available: Study Protocol, Statistical Analysis Plan (SAP), Informed Consent Form (ICF), Clinical Study Report (CSR), Analytic Code	vague+	vague+	vague+
NCT05007509	(As of February 2022): To be decided	no plans	no plans	no plans
NCT05027217	(As of March 2022): SANDMAN investigators will be given priority to lead secondary analyses and are encouraged to do so. Participation and authorship opportunities will be based on contribution to the primary study. The study steering committee will consider the scientific validity and the possible effect on the anonymity of participating centres prior to granting any such requests. Where necessary, a prior written agreement may be requested to set out the terms of such collaborations. Investigators should submit a secondary study proposal for review by the Executive committee. ‘Cleaned’ data from the international or national datasets will only be released after a secondary study proposal has been approved. An analysis involving any data derived from the SANDMAN study will be considered a secondary analysis and subject to these rules.<br>Time frame: After the main study report is published.<br>Access Criteria: Upon request to the study Executive Committee<br>URL: https://www.esicm.org/wp-content/uploads/2020/01/SANDMAN-Authorship-and-publication-Jan-22-2020.pdf<br>Information available: Study Protocol, Statistical Analysis Plan (SAP)	complex+	unclear	unclear
NCT05038488	(As of November 2021): Individual participant data will not be shared	no sharing	no sharing	no sharing

NCT05042063	(As of September 2021): Datasets with anonymized IPD, including cough registries and VAS scores will be shared at the end of the study.<br>Time frame: Data will become available at the completion of the study (2026) and will remain available from that moment onward.<br>Access Criteria: Upon request to researchers<br>Information available: Study Protocol, Informed Consent Form (ICF), Clinical Study Report (CSR)	request+	request+	request+
NCT05050682	(As of May 2022): Pfizer will provide access to individual de-identified participant data and related study documents (e.g. protocol, Statistical Analysis Plan (SAP), Clinical Study Report (CSR)) upon request from qualified researchers, and subject to certain criteria, conditions, and exceptions. Further details on Pfizer's data sharing criteria and process for requesting access can be found at: https://www.pfizer.com/science/clinical_trials/trial_data_and_results/data_requests.	complex+	request+	complex+
NCT05060861	(As of September 2021): upon reasonable request	request+	request+	request+
NCT05075941	(As of October 2021): The results will be presented in a report to the Ministry of Health, the Haute Autorité de Santé and the various partners. The main findings of this study could potentially be compared with the findings of other ongoing studies, once they are completed. An abstract of the findings will be submitted for presentation at a scientific conference, and at least one manuscript will be submitted for publication in a peer-reviewed scientific journal.<br>Access Criteria: Study partners<br>Information available: Study Protocol, Statistical Analysis Plan (SAP), Informed Consent Form (ICF), Clinical Study Report (CSR)	unclear	no sharing	no sharing

NCT05078905	(As of April 8, 2022): .Identified data in BTRIS (automatic for activities in the Clinical Center).@@@@@@De-identified or identified data with approved outside collaborators under appropriate agreements.@@@@@@This study will comply with the NIH Genomic Data Sharing Policy, which applies to all NIH-funded research that generates large-scale human or non-human genomic data, as well as the use of these data for subsequent research. Large-scale data include genome-wide association studies (GWAS), single nucleotide polymorphisms (SNP) arrays, and genome sequence, transcriptomic, epigenomic, and gene expression data. De-identified data may be shared in an NIH-funded or approved public repositories, including the Database of Genotypes and Phenotypes (dbGAP) or in NCBI Virus Portal for viral sequencing.<br>Time frame: Shared scientific data should be made accessible as soon as possible, and no later than the time of an associated publication, or the end of the award/support period, whichever comes first.@@@@@@Genomic data will be shared following the guidelines of the Genomic Data Sharing Policy, when applicable.<br>Access Criteria: Identified data in BTRIS (automatic for activities in the Clinical Center and will be available for use following the BTRIS Policy for Data Sharing and Use).@@@@@@De-identified or identified data with approved outside collaborators under appropriate agreements.@@@@@@De-identified genomic data may be shared in an NIH-funded or approved public repositories, including the Database of Genotypes and Phenotypes (dbGAP) and the use of the data within will be governed by their policies.@@@@@@01<br>Information available: Study Protocol	complex+	vague+	complex+
NCT05083130	(As of April 2022): Anonymised data of this study may be requested for publication by journals. Sharing anonymised data with future similar/suitable studies will be decided by the sponsor, PIs and the authority agency where the data was collected. No identifiable information will be shared with any other person/organisation<br>Information available: Study Protocol	vague+	vague+	vague+

NCT05085613	(As of December 2021): Upon request, deidentified participant data collected during the study may be shared. Upon request, we may also share the study protocol, survey, and informed consent form. Data will be made available upon request beginning 3 months following publication of the final article from this study, with no end date. Data will be made available for analyses deemed appropriate by the study PI. Proposals should be directed to mmoran22@jhu.edu. Data requestors will need to sign a data access agreement.<br>Time frame: Starting 3 months following publication of the final article from this study, with no end date.<br>Information available: Study Protocol, Informed Consent Form (ICF)	request+	complex+	request+
NCT05115617	(As of November 2021): IPD that underlie the results reported in this article, after deidentification, will be made available to other researchers upon request.	request+	request+	request+
NCT05148819	(As of December 2021): anonymous questionnaire to evaluate the effect of COVID-19 recovered patients on their sexual function.	vague+	unclear	unclear
NCT05165953	(As of December 2021): all collected data<br>Time frame: after acceptance by journal, for 1 year<br>Information available: Study Protocol, Statistical Analysis Plan (SAP), Clinical Study Report (CSR)	vague+	vague+	vague+

NCT05181683	(As of February 2022): All Individual Patient Data (IPD) that underlie publicly available results will be considered for sharing<br>Time frame: When Regeneron has received marketing authorization from major health authorities (e.g., FDA, European Medicines Agency (EMA), Pharmaceuticals and Medical Devices Agency (PMDA), etc.) for the product and indication, has made the study results publicly available (e.g., scientific publication, scientific conference, clinical trial registry), has the legal authority to share the data, and has ensured the ability to protect participant privacy.<br>Access Criteria: Qualified researchers can submit a proposal for access to individual patient or aggregate level data from a Regeneron-sponsored clinical trial through Vivli. Regeneron's Independent Research Request Evaluation Criteria can be found at: https://www.regeneron.com/sites/default/files/Regeneron-External-Data-Sharing-Policy-and-Independent-Research-Request-Evaluation-Criteria.pdf<br>URL: https://vivli.org/<br>Information available: Study Protocol, Statistical Analysis Plan (SAP), Informed Consent Form (ICF), Clinical Study Report (CSR), Analytic Code	storage+	storage+	storage+
NCT05225298	(As of February 2022): Anonymized data will be shared with NIH Radx-UP data repository.<br>Information available: Study Protocol, Statistical Analysis Plan (SAP), Clinical Study Report (CSR)	storage+	storage+	storage+
NCT05231005	(As of February 2022): Unidentified data will be available after publication of the results (all IPD that underlie results in a publication)<br>Time frame: will be available upon publication of results.<br>Access Criteria: upon request<br>Information available: Study Protocol, Statistical Analysis Plan (SAP)	request+	request+	request+
NCT05255848	(As of April 2022): Planned international meta-trial see NCT04635241<br>Time frame: Real-Time<br>Information available: Study Protocol, Statistical Analysis Plan (SAP), Informed Consent Form (ICF), Clinical Study Report (CSR), Analytic Code	unclear	unclear	unclear

NCT05263908	(As of May 2022): Pfizer will provide access to individual de-identified participant data and related study documents (e.g. protocol, Statistical Analysis Plan (SAP), Clinical Study Report (CSR)) upon request from qualified researchers, and subject to certain criteria, conditions, and exceptions. Further details on Pfizer's data sharing criteria and process for requesting access can be found at: https://www.pfizer.com/science/clinical_trials/trial_data_and_results/data_requests.	complex+	request+	complex+
NCT05305651	(As of March 2022): IPD for this study will be made available via the Clinical Study Data Request site.<br>Time frame: IPD will be made available within 6 months of publishing the results of the primary endpoints, a key secondary endpoints and safety data of the study.<br>Access Criteria: Access is provided after a research proposal is submitted and has received approval from the Independent Review Panel and after a Data Sharing Agreement is in place. Access is provided for an initial period of 12 months but an extension can be granted, when justified, for up to another 12 months.<br>URL: http://clinicalstudydatarequest.com<br>Information available: Study Protocol, Statistical Analysis Plan (SAP), Informed Consent Form (ICF), Clinical Study Report (CSR)	complex+	storage+	storage+
NCT05327426	(As of April 2022): Aggregated data will be made available to other researchers.	vague+	no sharing	no sharing

NCT05336565	(As of June 2022): eidentified individual participant data (text, tables, figures, and appendices), underlying the results of the trial, will be shared with researchers to achieve the aims in the approved proposal.<br>Time frame: Proposals may be submitted up to 36 months following publication of the results of the trial. After 36 months, the data will be available in the Center's data ware house but without investigator support other than deposited metadata.<br>Access Criteria: Information regarding submitting proposals and accessing data may be requested from the principal investigator by e-mail.<br>Information available: Study Protocol, Statistical Analysis Plan (SAP), Clinical Study Report (CSR)	request+	request+	request+
NCT05378191	(As of May 2022): Will submit data to European Union Drug Regulating Authorities Clinical Trials Database (EudraCT) and share data result.<br>Time frame: Within one year after study end.<br>Access Criteria: Published in EudraCT<br>URL: https://eudract.ema.europa.eu/index.html<br>Information available: Study Protocol, Statistical Analysis Plan (SAP), Informed Consent Form (ICF), Clinical Study Report (CSR)	storage+	unclear	unclear
NCT05385029	(As of May 2022): After statistical analysis all data will be shared for discussion and final approval<br>Time frame: 3 moths<br>Information available: Study Protocol, Statistical Analysis Plan (SAP), Clinical Study Report (CSR)	vague+	vague+	vague+
NCT05394961	(As of May 2022): Individual participant data are used with special permission from the national ethical bord of Sweden (Etikprövningsmyndigheten) and the involved register keepers and so cannot be shared without an extended permission.	complex+	no sharing	unclear
NCT05398848	(As of May 2022): There is not a plan to make individual participant data (IPD) available	no sharing	no plans	no sharing

PACTR202006922165132	The data accumulated from this study will be made publically available, within one year of completion of the study to any investigators or BMGF nominated partners, who wish to use the data to address any specific questions not directly addressed under the study objectives and which the data would lend itself to. Any transfer of data, will be governed, by the terms of the local Ethics Committee (HREC). Data sharing will be done on a collaborative basis, with the site PI (or his nominee) being included in any further interrogation of the data. The data will be provided in the format in which it has been entered at RMPRU with the necessary data dictionary.	complex+	complex+	complex+
PACTR202009769021840	Individual participant data that underlie the result of the study after deidentification	vague+	vague+	vague+
PACTR202102616421588	All of the individual participant data collected during the trial, after deidentification, will be posted publicly once the main manuscript using the endline data has been published. The study protocol, statistical analysis plan, informed consent form, and analytic code will also be available.	vague+	vague+	vague+
PACTR202103845381761	Data collected from this study, including de-identified individual participant data, will be made available upon publication to members of the scientific and medical community for non-commercial use only, upon email request to the corresponding author.	request+	request+	request+
PACTR202106466447805	ClinicalTrials.gov Identifier: NCT04372186 NEJM publication link - https://pubmed.ncbi.nlm.nih.gov/33332779/	unclear	unclear	unclear

PACTR202203469339247	Your study records will be identified by a unique study number. This number will not include any personal information that could identify you (i.e. it will not include your name, Personal Health Number, Social Insurance Number, or initials, etc.). The number will be used on any research-related information and samples that go outside the study site. The list that matches your name to the unique study number that is used on research-related information is kept at the study site securely and will not be removed and/or released without your consent, unless required by law. Your study records, including confidential information collected during the study, will be kept in a secure location for at least 15 years. Study information, your study code, and samples collected as part of this study will be included in secure electronic trial systems. These systems may be managed and monitored by companies who work with VIDO. No information that discloses your identity will be released or published without your specific consent. Rarely, your study documents may be obtained by courts of law. Unless required by law, your name will not be disclosed outside the research clinic. Your name will be available only to the following people or agencies: the study doctor and staff; and authorized representatives of the study doctor; the Kenyatta National Hospital – University of Nairobi Ethics and Research Committee (KNH/UoN ERC), the Pharmacy and Poisons Board (PPB), VIDO-authorized representatives; and authorized Clinical Research Organization representatives. The above-mentioned individuals as well as some authorities will use the personal information collected as part of this study, which may include your medical records so as to check that the study is conducted correctly and to ensure your study and medical record information is correct. These people are all obligated to maintain confidentiality by the nature of their work, or are bound by confidentiality agreements. Your study	unclear	unclear	unclear
PACTR202205492711803	Individual participant data that underlie the results reported in journal publication, after deidentification	vague+	vague+	vague+
RBR-33ndb47	The data will be made available at ARCA Dados/ Fiocruz upon request including an anonymized individual patients (IPD) dataset; data dictionary and electronic CRF audit logs.	request+	request+	request+
TCTR20200822002	published journal	vague+	no sharing	no sharing

TCTR20210412005	Need a consensus from colleagues	no plans	no plans	no plans
TCTR20210802006	We did not request for permission to share data from the participants to public	no sharing	no sharing	no sharing
TCTR20210909002	After published	vague+	vague+	vague+
TCTR20210923012	Subjects' confidentiality	unclear	no sharing	no sharing
TCTR20211005002	Prohibit from regulation	no sharing	no sharing	no sharing
TCTR20220317003	Need a consensus from the participant	unclear	no plans	no plans
TCTR20220330001	need a consensus from colleagues	no plans	no plans	no plans
TCTR20220402001	IPD and documents will be available for sharing after publication.	vague+	vague+	vague+
TCTR20220407003	Need a consensus from colleagues	no plans	no plans	no plans
TCTR20220524004	Deidentified participant data and study protocol are available from the study director upon reasonable requests made via email. The data will be available immediately following publication without end date	request+	request+	request+
